# Supplementary material for: Evolution of the HIV-1 integration site landscape and inducible reservoir in early-treated people
Source: PLoS Pathog. 2025 Nov 25;21(11):e1013702. doi: 10.1371/journal.ppat.1013702 (PMC12646413; doi:10.1371/journal.ppat.1013702)
Supplement: S1 Table — Fiebig stage at the time of diagnosis is indicated. The table shows which samples from each participant (‘x’) were used in the different assays. MIP-Seq ISLA refers to ISLA performed on MDA-amplified material, while ISLA refers to ISLA performed on bulk genomic DNA. VL = viral load; ART = antiretroviral therapy; UD = undetectable; MIP-Seq = Matched Integration site and Proviral Sequencing; ISLA = Integration Site Loop Amplification; HG = Half Genomes; FLIPS = Full-Length Individual Proviral Sequencing; HIV-PULSE = HIV Proviral UMI-mediated Long-read Sequencing; STIP-Seq = Simultaneous TCR, Integration site and Proviral sequencing. (PDF) [file ppat.1013702.s008.pdf]

Supplementary Table 1: Clinical characteristics of participants.

Participant ID	Age (years)	Gender	Subtype	VL	Time since infection (years)	Time to ART (months)	Time to UD VL (years)	ART duration (years)	Cohort	Fiebig group	Rainbow	MIP-Seq ISLA	MIP-Seq HG	ISLA	FLIPS	HIV-PULSE	HIV-flow	STIP-Seq
PA01	44	M	B	<20	1,9	1,1	0,3	1,8	Acute UD	Fiebig IV	x							
PA02	31	M	B	<20	2,0	0,6	1,1	1,9	Acute UD	Fiebig III	x	x	x				x	x
PA02	36	M	B	<20	7,5	0,6	1,1	7,4	Acute UD+5	Fiebig III	x			x	x		x	
PA03	37	M	CRF02_AG	<20	1,5	0,7	1,4	1,4	Acute UD	Fiebig V	x							
PA04	51	M	CRF02_AG	<20	1,7	4,9	0,6	1,3	Acute UD	Fiebig V	x							
PA05	31	M	B	<20	1,7	1,4	1,4	1,6	Acute UD	Fiebig II	x	x	x				x	
PA05	36	M	B	<20	7,1	1,4	1,4	7,0	Acute UD+5	Fiebig II	x			x	x		x	
PA06	40	M	B	<20	1,1	1,8	0,6	0,9	Acute UD	Fiebig V	x							
PA07	44	M	/	<20	0,8	0,6	0,4	0,7	Acute UD	Fiebig V	x							
PA08	42	M	/	<20	1,8	4,2	0,5	1,4	Acute UD	Fiebig V	x							
PA09	31	M	/	<20	0,6	1,6	0,4	0,4	Acute UD	Fiebig III	x							
PA10	31	M	B	<20	1,0	1,4	0,4	0,9	Acute UD	Fiebig V	x							
PA11	54	M	/	<20	0,9	0,6	0,5	0,8	Acute UD	Fiebig III	x							
PA12	30	M	B	<20	0,9	1,7	0,3	0,8	Acute UD	Fiebig II	x						x	
PA13	32	M	CRF02_AG	<20	/	/	0,5	0,5	Acute UD	Fiebig III	x							
PA14	36	M	B	<20	1,1	1,5	0,7	1,0	Acute UD	Fiebig V	x	x	x				x	x
PA15	40	M	B	<20	1,3	4,7	0,3	0,9	Acute UD	Fiebig V	x	x	x				x	
PA15	44	M	B	<20	5,7	4,7	0,3	5,3	Acute UD+5	Fiebig V	x			x	x		x	
PA16	31	M	C	<20	0,5	5,4	0,1	0,1	Acute UD	Fiebig VI	x							
PA17	33	M	F1	<20	1,1	0,9	1,0	1,0	Acute UD	Fiebig III	x							
PA18	37	M	/	<20	0,5	0,3	0,3	0,5	Acute UD	Fiebig II	x							
PA19	40	M	B	<20	0,8	4,2	0,2	0,5	Acute UD	Fiebig V	x	x	x				x	
PA19	44	M	B	<20	5,2	4,2	0,2	4,9	Acute UD+5	Fiebig V	x			x	x		x	
PA20	30	M	UD	<20	0,8	1,0	0,3	0,8	Acute UD	Fiebig II	x							
PA21	63	M	B	<20	1,6	2,1	0,5	1,5	Acute UD	Fiebig II	x							
PA22	38	F	F1	<20	1,1	1,3	0,4	1,0	Acute UD	Fiebig VI	x							
PA23	31	M	B	<20	1,0	1,5	0,6	0,9	Acute UD	Fiebig V	x							
PA24	20	M	B	<20	1,2	2,5	0,3	1,0	Acute UD	Fiebig II	x							
PA25	27	M	B	<20	0,8	1,5	0,2	0,7	Acute UD	Fiebig V	x							
PA26	30	M	B	<20	0,7	1,5	2,0	0,6	Acute UD	Fiebig V	x							
PA27	42	F	B	<20	1,0	6,3	0,2	0,5	Acute UD	Fiebig V	x							
PA28	41	M	B	<20	0,8	0,9	1,3	0,8	Acute UD	Fiebig III	x							
PA29	55	M	F1	<20	/	/	0,5	0,7	Acute UD	Fiebig V	x							
PA30	62	M	C	<20	0,4	0,9	0,3	0,3	Acute UD	Fiebig II	x							
PA31	33	M	CRF02_AG	<20	0,4	1,4	0,1	0,3	Acute UD	Fiebig V	x							
PA32	29	M	B	<20	0,5	1,2	0,4	0,4	Acute UD	Fiebig V	x							
PA33	52	M	A	<20	0,7	1,7	0,5	0,5	Acute UD	Fiebig V	x							
PA34	56	M	B	<20	0,5	1,0	0,2	0,4	Acute UD	Fiebig V	x	x	x				x	x
PA35	44	M	B	<20	0,5	0,8	0,3	0,5	Acute UD	Fiebig II	x	x	x				x	x
PA35	47	M	B	<20	3,5	0,8	0,3	3,4	Acute UD+5	Fiebig II	x			x	x		x	
PA36	46	M	A	<20	0,9	1,6	0,4	0,7	Acute UD	Fiebig V	x							
PA37	45	F	CRF02_AG	<20	1,1	1,7	1,0	1,0	Acute UD	Fiebig V	x							
PA38	27	M	B	<20	0,4	1,2	0,1	0,3	Acute UD	Fiebig II		x	x				x	
PA39	62	M	F1	<20	1,2	2,7	0,3	1,0	Acute UD	Fiebig IV	x							
PC01	27	M	F1	<20	2,9	31	0,2	0,3	Chronic UD	/	x						x	
PC02	42	M	B	<20	1,7	16	0,3	0,3	Chronic UD	/	x			x	x		x	
PC03	27	M	CRF02_AG	<20	0,7	4	0,2	0,4	Chronic UD	/	x						x	
PC04	34	M	B	<20	1,5	15	0,1	0,2	Chronic UD	/	x			x	x		x	
PC05	49	M	CRF02_AG	<20	1,1	8	0,3	0,5	Chronic UD	/	x						x	
PC06	27	F	/	<20	1,1	3	0,6	0,9	Chronic UD	/	x						x	
PC07	31	M	A6	<20	2,2	19	0,4	0,6	Chronic UD	/	x						x	
PC08	45	M	F1	<20	1,1	9	0,1	0,4	Chronic UD	/	x						x	
PC09	36	M	CRF02_AG	<20	2,1	9	0,2	1,3	Chronic UD	/	x						x	
PC10	40	M	B	<20	2,7	30	0,1	0,3	Chronic UD	/	x			x	x		x	
PC11	55	M	B	<20	/	/	/	18,5	Chronic UD+x	/	x					x	x	
PC12	51	M	B	<20	/	/	/	18,4	Chronic UD+x	/	x					x	x	
PC13	37	M	B	<20	7,5	29,1	/	5,1	Chronic UD+x	/	x					x	x	
PC14	50	M	B	<20	16,5	182,4	/	1,3	Chronic UD+x	/	x					x	x	
PC15	56	M	B	<20	/	/	/	14,7	Chronic UD+x	/	x					x	x	
PC16	41	M	B	<20	/	/	/	7,6	Chronic UD+x	/	x						x	
PC17	49	M	B	<20	19,8	40,8	/	16,4	Chronic UD+x	/	x					x	x	
PC18	61	M	B	<20	30,6	69,2	/	24,8	Chronic UD+x	/	x					x	x	
PC19	54	M	B	<20	15,6	34,0	/	12,8	Chronic UD+x	/	x					x	x	
PC20	48	M	B	<20	13,6	31,1	/	11,1	Chronic UD+x	/	x					x	x	
PC21	/	/	B	<20	/	/	/	/	Chronic UD+x	/	x						x	
PC22	62	M	B	<20	/	/	0,3	4,4	Chronic UD+x	/	x							
PC23	42	M	B	<20	13	83,2	/	6,1	Chronic UD+x	/	x							
PC24	50	F	C	<20	/	/	0,2	3,7	Chronic UD+x	/	x							
PC25	53	F	A1	<20	/	/	/	17,1	Chronic UD+x	/	x							
PC26	37	F	A1	<20	14,2	46,4	/	10,4	Chronic UD+x	/	x							
PC27	40	M	B	<20	8,4	12,7	/	7,3	Chronic UD+x	/	x							
PC28	31	M	B	<20	11,7	50,0	/	7,5	Chronic UD+x	/	x							
PC29	26	M	Recomb B/F1	<20	/	/	0,6	7,2	Chronic UD+x	/	x							
PC30	61	M	CRF02_AG	<20	11	61,7	0,3	5,9	Chronic UD+x	/	x							
PC31	49	F	F1	<20	17,4	33,9	/	14,6	Chronic UD+x	/	x							
PC32	46	M	B	<20	/	/	/	6,7	Chronic UD+x	/	x							
PC33	32	M	B	<20	/	/	0,3	1,4	Chronic UD+x	/	x							
PC34	62	M	B	<20	11,6	35,7	0,5	8,7	Chronic UD+x	/	x							
PC35	58	M	B	<20	17	8,3	/	16,3	Chronic UD+x	/	x							
PC36	/	/	B	<20	/	/	/	/	Chronic UD+x	/	x							
PC37	/	/	B	<20	/	/	/	/	Chronic UD+x	/	x							
PC38	/	/	B	<20	/	/	/	/	Chronic UD+x	/	x							
PC39	/	/	B	<20	/	/	/	/	Chronic UD+x	/	x							
PC40	/	/	B	<20	/	/	/	/	Chronic UD+x	/	x							
PC41	68	M	B	<20	6,5	43	0,2	2,9	Chronic UD+x	/	x							
PC42	47	M	B	<20	10,8	89	0,0	3,3	Chronic UD+x	/	x							
PC43	46	M	B	<20	11,3	7	7,3	10,8	Chronic UD+x	/	x							
PC44	27	M	B	<20	5,6	13	0,1	4,5	Chronic UD+x	/	x							
PC45	45	M	B	<20	10,0	63	0,2	4,8	Chronic UD+x	/	x							
PC46	41	M	B	<20	8,5	39	0,3	5,3	Chronic UD+x	/	x							
PC47	41	M	B	<20	5,7	5	0,2	5,3	Chronic UD+x	/	x							
PC48	47	M	B	<20	8,3	35	0,1	5,4	Chronic UD+x	/	x							
PC49	46	M	B	<20	7,3	17	0,4	5,9	Chronic UD+x	/	x							
PC50	45	M	B	<20	7,8	13	0,7	6,8	Chronic UD+x	/	x							
PC51	62	M	B	<20	7,1	12	0,0	6,1	Chronic UD+x	/	x							
PC52	60	M	B	<20	9,9	45	0,1	6,2	Chronic UD+x	/	x							
PC53	45	M	B	<20	11,9	44	0,3	8,3	Chronic UD+x	/	x							
PC54	67	M	B	<20	9,1	6	0,3	8,6	Chronic UD+x	/	x							
PC55	42	M	B	<20	8,9	5	0,2	8,5	Chronic UD+x	/	x							
PC56	56	M	B	<20	31,2	261	0,1	9,4	Chronic UD+x	/	x							
PC57	32	M	B	<20	14,2	51	0,3	9,9	Chronic UD+x	/	x							
PC58	47	M	B	<20	11,3	15	0,2	10,0	Chronic UD+x	/	x							
PC59	39	M	B	<20	11,5	15	0,4	10,3	Chronic UD+x	/	x							
PC60	36	M	B	<20	13,3	38	0,2	10,2	Chronic UD+x	/	x							
PC61	39	M	B	<20	13,3	34	0,3	10,5	Chronic UD+x	/	x							
PC62	49	M	B	<20	16,2	61	0,6	11,1	Chronic UD+x	/	x							
PC63	67	M	B	<20	12,2	13	0,4	11,1	Chronic UD+x	/	x							
PC64	51	M	B	<20	19,1	94	0,3	11,3	Chronic UD+x	/	x							
PC65	41	M	B	<20	16,7	53	0,3	12,3	Chronic UD+x	/	x							

PC66	48	M	B	<20	15,8	18	0,6	14,3	Chronic UD+x	/	x							
PC67	52	M	B	<20	15,9	14	0,6	14,8	Chronic UD+x	/	x							
