# Supplementary material for: Evolution of the HIV-1 integration site landscape and inducible reservoir in early-treated people
Source: PLoS Pathog. 2025 Nov 25;21(11):e1013702. doi: 10.1371/journal.ppat.1013702 (PMC12646413; doi:10.1371/journal.ppat.1013702)
Supplement: S2 Table — Integration sites were mapped to the GRCh38.p14 human genome reference assembly. The column Strand refers to genome orientation: strand = F indicates that HIV-1 is on the forward strand, while strand = R indicates reverse strand. The column Orientation refers to gene orientation: Same indicates that HIV-1 has the same orientation relative to the gene where it integrated, while Opposite indicates HIV-1 has the opposite orientation relative to the gene where it is integrated. Integration sites found more than once are marked by ‘x’ in the column Clonal. HIV-1 integrated in a ZNF KRAB gene on chromosome 19 is marked by ‘x’ in the column ZNF KRAB chr19. Sequence nr. = Sequence number; ISLA = Integration Site Loop Amplification; NA = not available; chr = chromosome. (PDF) [file ppat.1013702.s009.pdf]

Supplementary Table 2. List of integration sites

Cohort	Participant ID	Assay	Sequence nr.	Chromosome	Position	Strand	Gene	Orientation	Repetitive sequence	Clonal	ZNF KRAB chr19	Hi-C compartment
Chronic UD	PC10	ISLA total CD4 T cells	1	2	221552151	F	EPHA4	Opposite	NA			B3
Chronic UD	PC10	ISLA total CD4 T cells	2	19	46519404	R	PPP5D1	Same	NA			B1
Chronic UD	PC10	ISLA total CD4 T cells	3	4	87011538	R	AFF1	Opposite	NA			B3
Chronic UD	PC10	ISLA total CD4 T cells	4	1	107833227	F	VAV3	Opposite	Name: Tigger1; Class: DNA; Family: TcMar-Tigger			B3
Chronic UD	PC10	ISLA total CD4 T cells	5	4	98613920	F	TSPAN5	Opposite	NA			B3
Chronic UD	PC10	ISLA total CD4 T cells	6	16	28563534	R	CCDC101	Opposite	NA			A1
Chronic UD	PC10	ISLA total CD4 T cells	7	11	65669585	F	LOC105369347	Same	Name: L1M4; Class: LINE; Family: L1			A1
Chronic UD	PC10	ISLA total CD4 T cells	8	6	25989268	R	Upstream: TRIM38 (1.939 kb); Downstream: HIST1H1PS2 (26.839 kb)		Name: L1MC1; Class: LINE; Family: L1			A2
Chronic UD	PC10	ISLA total CD4 T cells	9	16	29706820	R	Upstream: QPRT (8.826 kb); Downstream: C16orf54 (35.643 kb)		Name: AluY; Class: SINE; Family: Alu			A1
Chronic UD	PC10	ISLA total CD4 T cells	10	8	144299196	F	HSF1	Same	Name: AluSx1; Class: SINE; Family: Alu			A1
Chronic UD	PC10	ISLA total CD4 T cells	11	1	43758643	R	ST3GAL3	Opposite	Name: L1MEd; Class: LINE; Family: L1			A1
Chronic UD	PC10	ISLA total CD4 T cells	12	4	464147	F	ABCA11P	Opposite	Name: L1M3a; Class: LINE; Family: L1			B1
Chronic UD	PC10	ISLA total CD4 T cells	13	4	464147	F	ZNF721	Opposite	Name: L1M3a; Class: LINE; Family: L1			B1
Chronic UD	PC10	ISLA total CD4 T cells	14	17	2267240	R	SMG6	Same	Name: AluJb; Class: SINE; Family: Alu			A1
Chronic UD	PC10	ISLA total CD4 T cells	15	16	29016373	R	Upstream: LAT (25.590 kb); Downstream: LOC730153 (21.461 kb)		Name: L1M3; Class: LINE; Family: L1			A1
Chronic UD	PC10	ISLA total CD4 T cells	16	14	21922610	R	TRA	Opposite	NA			A1
Chronic UD	PC10	ISLA total CD4 T cells	17	3	121719108	F	GOLGB1	Opposite	NA			A2
Chronic UD	PC10	ISLA total CD4 T cells	18	17	78749889	R	CYTH1	Same	NA			A1
Chronic UD	PC10	ISLA total CD4 T cells	19	15	43444612	F	TP53BP1	Opposite	Name: MIR; Class: SINE; Family: MIR			A1
Chronic UD	PC10	ISLA total CD4 T cells	20	17	61230295	R	BCAS3	Opposite	NA	x		B1
Chronic UD	PC10	ISLA total CD4 T cells	21	17	61230295	R	BCAS3	Opposite	NA	x		B1
Chronic UD	PC10	ISLA total CD4 T cells	22	8	120591006	R	SNTB1	Same	NA			B3
Chronic UD	PC10	ISLA total CD4 T cells	23	9	94264248	R	ZNF169	Opposite	Name: AluSz; Class: SINE; Family: Alu			B1
Chronic UD	PC10	ISLA total CD4 T cells	24	17	81895768	F	ANAPC11	Same	Name: AluSc5; Class: SINE; Family: Alu			NA
Chronic UD	PC10	ISLA total CD4 T cells	25	11	66111298	R	PACS1	Opposite	Name: Tigger3a; Class: DNA; Family: TcMar-Tigger			A1
Chronic UD	PC10	ISLA total CD4 T cells	26	11	77707335	R	RSF1	Same	NA			A1
Chronic UD	PC10	ISLA total CD4 T cells	27	14	52653820	R	ERO1L	Same	NA			B1
Chronic UD	PC10	ISLA total CD4 T cells	28	6	108615400	F	FOXO3	Same	Name: L1ME1; Class: LINE; Family: L1			A2
Chronic UD	PC10	ISLA total CD4 T cells	29	17	78695602	F	CYTH1	Opposite	NA			A1
Chronic UD	PC10	ISLA total CD4 T cells	30	20	33844731	R	CHMP4B	Opposite	NA			A1
Chronic UD	PC10	ISLA total CD4 T cells	31	18	26056163	F	SS18	Opposite	NA			B2
Chronic UD	PC10	ISLA total CD4 T cells	32	22	38556761	F	DMC1	Opposite	NA			A1
Chronic UD	PC10	ISLA total CD4 T cells	33	4	26939915	R	STIM2	Opposite	NA			A2
Chronic UD	PC10	ISLA total CD4 T cells	34	1	22068672	R	CDC42	Mixed	NA			A1
Chronic UD	PC10	ISLA total CD4 T cells	34	1	22068672	R	LOC101928071					
Chronic UD	PC10	ISLA total CD4 T cells	35	10	11977577	F	UPF2	Opposite	NA			A2
Chronic UD	PC10	ISLA total CD4 T cells	36	3	123544292	F	HACD2	Opposite	NA			B1
Chronic UD	PC10	ISLA total CD4 T cells	37	5	163478652	R	HMMR	Opposite	NA			B3
Chronic UD	PC10	ISLA total CD4 T cells	38	2	181180651	F	LOC101927156	Same	NA			B3
Chronic UD	PC10	ISLA total CD4 T cells	39	16	89788259	F	FANCA	Opposite	Name: AluSc; Class: SINE; Family: Alu			A1
Chronic UD	PC10	ISLA total CD4 T cells	40	4	129064816	R	SCLT1	Same	Name: L1M5; Class: LINE; Family: L1	x		A2
Chronic UD	PC10	ISLA total CD4 T cells	41	4	129064816	R	SCLT1	Same	Name: L1M5; Class: LINE; Family: L1	x		A2
Chronic UD	PC10	ISLA total CD4 T cells	42	12	31981933	F	KIAA1551	Same	NA			A2
Chronic UD	PC10	ISLA total CD4 T cells	43	17	37629484	F	DDX52	Opposite	Name: AluJo; Class: SINE; Family: Alu			A1
Chronic UD	PC10	ISLA total CD4 T cells	44	2	201065045	F	FAM126B	Opposite	Name: L1MD2; Class: LINE; Family: L1			B3
Chronic UD	PC10	ISLA total CD4 T cells	45	17	28082379	R	NLK	Opposite	NA			A1
Chronic UD	PC10	ISLA total CD4 T cells	46	18	9141179	R	ANKRD12	Opposite	NA			A1
Chronic UD	PC10	ISLA total CD4 T cells	47	19	7397901	R	ARHGEF18	Opposite	NA			NA
Chronic UD	PC10	ISLA total CD4 T cells	48	19	21180276	R	ZNF431	Opposite	Name: L1PA10; Class: LINE; Family: L1		x	B2
Chronic UD	PC10	ISLA total CD4 T cells	49	17	7496422	R	POLR2A	Opposite	NA			A1
Chronic UD	PC10	ISLA total CD4 T cells	50	19	41560849	R	CEACAM21	Opposite	Name: L1ME3D; Class: LINE; Family: L1			B1
Chronic UD	PC10	ISLA total CD4 T cells	51	3	52261892	F	WDR82	Opposite	Name: L1MB7; Class: LINE; Family: L1			A1
Chronic UD	PC10	ISLA total CD4 T cells	52	19	10996030	R	SMARCA4	Opposite	NA			A1
Chronic UD	PC10	ISLA total CD4 T cells	53	16	29442780	R	SMG1P6	Same	Name: AluSq2; Class: SINE; Family: Alu			NA
Chronic UD	PC10	ISLA total CD4 T cells	53	16	29544545	R	SMG1P2	Same	Name: AluSq2; Class: SINE; Family: Alu			NA
Chronic UD	PC10	ISLA total CD4 T cells	54	20	13229377	R	ISM1	Opposite	Name: L1ME2; Class: LINE; Family: L1			B2
Chronic UD	PC10	ISLA total CD4 T cells	55	16	20830043	R	LOC81691	Opposite	NA			A1
Chronic UD	PC10	ISLA total CD4 T cells	56	X	136764668	R	ARHGEF6	Same	Name: L1ME3A; Class: LINE; Family: L1			NA
Chronic UD	PC10	ISLA total CD4 T cells	57	1	155368440	R	ASH1L	Same	Name: L1M5; Class: LINE; Family: L1			A1
Chronic UD	PC10	ISLA total CD4 T cells	58	7	128616286	R	LOC101928451	Opposite	NA			A1
Chronic UD	PC10	ISLA total CD4 T cells	59	17	51239794	F	MBTD1	Opposite	NA			B2
Chronic UD	PC10	ISLA total CD4 T cells	60	17	28048722	R	NLK	Opposite	NA			A1
Chronic UD	PC10	ISLA total CD4 T cells	61	17	28048722	R	LOC102724517	Opposite	NA			A1
Chronic UD	PC10	ISLA total CD4 T cells	62	19	46901786	F	Upstream: AP2S1 (50.791 kb); Downstream: ARHGAP35 (16.781 kb)		NA			B1
Chronic UD	PC10	ISLA total CD4 T cells	63	1	21065613	F	EIF4G3	Opposite	Name: MIRb; Class: SINE; Family: MIR			A1
Chronic UD	PC10	ISLA total CD4 T cells	64	10	63450039	F	JMJD1C	Opposite	Name: AluJr4; Class: SINE; Family: Alu			B3
Chronic UD	PC10	ISLA total CD4 T cells	65	10	92933249	R	EXOC6	Opposite	Name: L1MEd; Class: LINE; Family: L1			A2
Chronic UD	PC10	ISLA total CD4 T cells	66	8	99712738	R	VPS13B	Opposite	NA			A2
Chronic UD	PC10	ISLA total CD4 T cells	67	17	36521670	F	MYO19	Opposite	Name: HAL1; Class: LINE; Family: L1			A1
Chronic UD	PC10	ISLA total CD4 T cells	68	X	124724607	F	TENM1	Opposite	Name: AluJb; Class: SINE; Family: Alu			NA
Chronic UD	PC04	ISLA total CD4 T cells	69	3	47151596	F	SETD2	Opposite	Name: AluSx; Class: SINE; Family: Alu			A1
Chronic UD	PC04	ISLA total CD4 T cells	70	3	45733965	R	SACM1L	Opposite	NA	x		A1
Chronic UD	PC04	ISLA total CD4 T cells	71	3	45733965	R	SACM1L	Opposite	NA	x		A1
Chronic UD	PC04	ISLA total CD4 T cells	72	15	68294882	R	FEM1B	Opposite	NA			A1
Chronic UD	PC04	ISLA total CD4 T cells	73	19	21319011	F	ZNF708	Opposite	NA	x	x	B4
Chronic UD	PC04	ISLA total CD4 T cells	74	19	21319011	F	ZNF708	Opposite	NA	x	x	B4
Chronic UD	PC04	ISLA total CD4 T cells	75	19	21319011	F	ZNF708	Opposite	NA	x	x	B4
Chronic UD	PC04	ISLA total CD4 T cells	76	19	21319011	F	ZNF708	Opposite	NA	x	x	B4
Chronic UD	PC04	ISLA total CD4 T cells	77	20	48782951	F	PREX1	Opposite	Name: L2a; Class: LINE; Family: L2			A1
Chronic UD	PC04	ISLA total CD4 T cells	78	19	3158863	R	GNA15	Opposite	Name: AluSz; Class: SINE; Family: Alu			A1
Chronic UD	PC04	ISLA total CD4 T cells	79	17	5300109	F	RABEP1	Same	NA			A1
Chronic UD	PC04	ISLA total CD4 T cells	80	11	46771601	R	CKAP5	Same	Name: L1MD2; Class: LINE; Family: L1			A1
Chronic UD	PC04	ISLA total CD4 T cells	81	11	118774893	F	DDX6	Opposite	NA			A1
Chronic UD	PC04	ISLA total CD4 T cells	82	8	19748328	F	CSGALNACT1	Opposite	NA			B2
Chronic UD	PC04	ISLA total CD4 T cells	83	3	195307456	F	ACAP2	Opposite	NA			A1
Chronic UD	PC04	ISLA total CD4 T cells	84	17	42265323	R	STAT5B	Same	Name: L1PA3; Class: LINE; Family: L1			A1
Chronic UD	PC04	ISLA total CD4 T cells	85	15	91887901	R	SLCO3A1	Opposite	Name: MIRb; Class: SINE; Family: MIR			B1
Chronic UD	PC04	ISLA total CD4 T cells	86	19	47183118	R	SAE1	Opposite	Name: AluSx1; Class: SINE; Family: Alu			A1
Chronic UD	PC04	ISLA total CD4 T cells	87	17	45311245	F	MAP3K14	Opposite	NA			A1
Chronic UD	PC04	ISLA total CD4 T cells	88	17	1859604	R	RPA1	Opposite	Name: L1MEc; Class: LINE; Family: L1			A1
Chronic UD	PC04	ISLA total CD4 T cells	89	X	38877170	F	Upstream: MID1IP1 (70.638 kb); Downstream: LOC105373175 (380.931 kb)		NA			NA
Chronic UD	PC04	ISLA total CD4 T cells	90	15	61861364	R	LOC105370848	Mixed	Name: MER58A; Class: DNA; Family: hAT-Charlie			B1
Chronic UD	PC04	ISLA total CD4 T cells	90	15	61861364	R	VPS13C					
Chronic UD	PC04	ISLA total CD4 T cells	91	9	136912496	R	TRAF2	Opposite	Name: L1MC5a; Class: LINE; Family: L1			A1
Chronic UD	PC04	ISLA total CD4 T cells	92	16	67742639	F	RANBP10	Opposite	NA			A1
Chronic UD	PC04	ISLA total CD4 T cells	93	7	30359315	F	ZNRF2	Same	Name: L1M5; Class: LINE; Family: L1			A2
Chronic UD	PC04	ISLA total CD4 T cells	94	1	145257215	F	Upstream: PPIAL4D (15.016 kb); Downstream: RNVU1-14 (23.901 kb)		NA			B1
Chronic UD	PC04	ISLA total CD4 T cells	95	13	22126923	R	LOC105370108	Opposite	NA			A2
Chronic UD	PC04	ISLA total CD4 T cells	96	11	11862897	R	USP47	Opposite	Name: L1PB4; Class: LINE; Family: L1			A2
Chronic UD	PC04	ISLA total CD4 T cells	97	17	42268561	R	STAT5B	Same	NA			A1
Chronic UD	PC04	ISLA total CD4 T cells	98	2	37248687	R	NDUFAF7	Opposite	Name: AluSp; Class: SINE; Family: Alu			A2
Chronic UD	PC04	ISLA total CD4 T cells	99	16	31741404	R	ZNF720	Opposite	Name: L1PA16; Class: LINE; Family: L1			B2
Chronic UD	PC04	ISLA total CD4 T cells	100	7	129725919	R	NRF1	Opposite	NA			A2
Chronic UD	PC04	ISLA total CD4 T cells	101	11	67149332	R	KDM2A	Opposite	Name: MIRb; Class: SINE; Family: MIR			A1
Chronic UD	PC04	ISLA total CD4 T cells	102	19	29973355	F	URI1	Same	NA			B2
Chronic UD	PC04	ISLA total CD4 T cells	103	1	51262691	R	RNF11	Opposite	Name: AluSx; Class: SINE; Family: Alu			A2
Chronic UD	PC04	ISLA total CD4 T cells	104	16	50278045	R	ADCY7	Opposite	Name: L1ME4b; Class: LINE; Family: L1			A1
Chronic UD	PC04	ISLA total CD4 T cells	105	15	77427239	R	HMG20A	Opposite	NA			A1
Chronic UD	PC04	ISLA total CD4 T cells	106	15	41269517	F	CHP1	Same	Name: L2c; Class: LINE; Family: L2			A1
Chronic UD	PC04	ISLA total CD4 T cells	107	4	122295740	F	KIAA1109	Same	Name: L1PA11; Class: LINE; Family: L1			B3
Chronic UD	PC04	ISLA total CD4 T cells	108	17	75850754	F	WBP2	Opposite	Name: AluJb; Class: SINE; Family: Alu			A1
Chronic UD	PC04	ISLA total CD4 T cells	109	17	4125681	F	ZZEF1	Opposite	Name: L3; Class: LINE; Family: CR1			A1
Chronic UD	PC04	ISLA total CD4 T cells	110	14	34711472	R	CFL2	Same	NA			B2
Chronic UD	PC04	ISLA total CD4 T cells	111	12	120836596	F	SPPL3	Opposite	Name: MLT1H; Class: LTR; Family: ERVL-MaLR			A1
Chronic UD	PC04	ISLA total CD4 T cells	112	1	90931194	F	ZNF644	Opposite	NA			A2
Chronic UD	PC04	ISLA total CD4 T cells	113	9	127046057	F	RALGPS1	Same	Name: L1MC3; Class: LINE; Family: L1			A1
Chronic UD	PC04	ISLA total CD4 T cells	114	1	27908685	F	RPA2	Opposite	Name: AluSx; Class: SINE; Family: Alu			A1
Chronic UD	PC04	ISLA total CD4 T cells	115	X	41611156	R	CASK	Same	Name: MER20; Class: DNA; Family: hAT-Charlie			NA
Chronic UD	PC04	ISLA total CD4 T cells	116	1	160643852	F	SLAMF1	Opposite	Name: L1MA4; Class: LINE; Family: L1			A1
Chronic UD	PC04	ISLA total CD4 T cells	117	19	1363049	R	MUM1	Opposite	Name: L1MD2; Class: LINE; Family: L1			A1
Chronic UD	PC04	ISLA total CD4 T cells	118	9	125300138	F	GAPVD1	Same	NA			B1
Chronic UD	PC04	ISLA total CD4 T cells	119	14	58491110	F	KIAA0586	Same	NA			B2
Chronic UD	PC04	ISLA total CD4 T cells	120	15	44686312	F	PATL2	Opposite	Name: L1PA7; Class: LINE; Family: L1			A1
Chronic UD	PC04	ISLA total CD4 T cells	121	3	46291719	F	Upstream: CCR3 (25.013 kb); Downstream: UQCRC2P1 (18.674 kb)		Name: L1M5; Class: LINE; Family: L1			A1
Chronic UD	PC04	ISLA total CD4 T cells	122	X	150644649	F	MTM1	Same	NA			NA
Chronic UD	PC04	ISLA total CD4 T cells	123	1	31029713	R	PUM1	Same	NA			B1
Chronic UD	PC04	ISLA total CD4 T cells	124	14	101866319	R	PPP2R5C	Opposite	NA			B1
Chronic UD	PC04	ISLA total CD4 T cells	125	15	43069169	F	UBR1	Opposite	Name: MIRc; Class: SINE; Family: MIR			A1
Chronic UD	PC04	ISLA total CD4 T cells	126	16	53445029	F	RBL2	Same	NA			A2
Chronic UD	PC04	ISLA total CD4 T cells	127	19	36903507	F	ZNF829	Opposite	Name: LTR37-int; Class: LTR; Family: ERV1		x	B4
Chronic UD	PC04	ISLA total CD4 T cells	128	1	110375380	R	SLC16A4	Same	NA			A1
Chronic UD	PC04	ISLA total CD4 T cells	129	11	66127372	F	PACS1	Same	NA			A1
Chronic UD	PC04	ISLA total CD4 T cells	130	17	7880515	F	NAA38	Opposite	NA			A1
Chronic UD	PC04	ISLA total CD4 T cells	131	8	17981878	F	PCM1	Same	Name: L1ME4a; Class: LINE; Family: L1			B2
Chronic UD	PC04	ISLA total CD4 T cells	132	X	108121402	F	ATG4A	Same	NA			NA
Chronic UD	PC02	ISLA total CD4 T cells	133	9	137624657	R	EHMT1	Opposite	Name: AluJb; Class: SINE; Family: Alu			B1
Chronic UD	PC02	ISLA total CD4 T cells	134	11	72966620	F	FCHSD2	Opposite	NA			A1
Chronic UD	PC02	ISLA total CD4 T cells	135	12	48952836	F	ARF3	Opposite	NA			B1
Chronic UD	PC02	ISLA total CD4 T cells	136	17	80787511	R	RPTOR	Opposite	Name: L1MEh; Class: LINE; Family: L1			A1
Chronic UD	PC02	ISLA total CD4 T cells	137	4	38645527	F	KLF3-AS1	Opposite	Name: AluSz; Class: SINE; Family: Alu			A2
Chronic UD	PC02	ISLA total CD4 T cells	138	6	42803544	R	GLTSCR1L	Opposite	NA			A1
Chronic UD	PC02	ISLA total CD4 T cells	139	3	113000188	F	GTPBP8	Same	Name: AluSx; Class: SINE; Family: Alu			A2
Chronic UD	PC02	ISLA total CD4 T cells	140	12	45891352	F	ARID2	Same	NA			B2
Chronic UD	PC02	ISLA total CD4 T cells	141	16	29565463	R	SMG1P2	Same	NA			NA

Chronic UD	PC02	ISLA total CD4 T cells	142	16	79606421	R	LOC101928230	Opposite	NA			B2
Chronic UD	PC02	ISLA total CD4 T cells	143	6	35879112	R	SRPK1	Same	Name: L1M4c; Class: LINE; Family: L1			A1
Chronic UD	PC02	ISLA total CD4 T cells	144	2	20273664	R	PUM2	Same	NA			A2
Chronic UD	PC02	ISLA total CD4 T cells	145	16	11849063	F	RSL1D1	Opposite	Name: AluSz6; Class: SINE; Family: Alu			A1
Chronic UD	PC02	ISLA total CD4 T cells	146	12	110292363	R	ATP2A2	Opposite	Name: AluSz; Class: SINE; Family: Alu			A1
Chronic UD	PC02	ISLA total CD4 T cells	147	19	889147	F	MED16	Opposite	NA			A1
Chronic UD	PC02	ISLA total CD4 T cells	148	5	177345034	R	LMAN2	Same	NA			B1
Chronic UD	PC02	ISLA total CD4 T cells	149	19	37356236	R	HKR1	Opposite	Name: L1PA17; Class: LINE; Family: L1			B4
Chronic UD	PC02	ISLA total CD4 T cells	150	X	135358014	R	ZNF449	Opposite	Name: L1MB8; Class: LINE; Family: L1			NA
Chronic UD	PC02	ISLA total CD4 T cells	151	1	174832938	R	RABGAP1L	Opposite	Name: MIR; Class: SINE; Family: MIR			A2
Chronic UD	PC02	ISLA total CD4 T cells	152	10	89280903	F	LOC105378419	Opposite	Name: LTR81A; Class: LTR; Family: Gypsy			A2
Chronic UD	PC02	ISLA total CD4 T cells	153	6	90030517	R	BACH2	Same	Name: L1PA4; Class: LINE; Family: L1			A2
Chronic UD	PC02	ISLA total CD4 T cells	154	5	55962775	F	IL6ST	Opposite	NA			A2
Chronic UD	PC02	ISLA total CD4 T cells	155	15	63727642	F	HERC1	Opposite	NA			A1
Chronic UD	PC02	ISLA total CD4 T cells	156	22	40529958	F	MKL1	Opposite	NA			A1
Chronic UD	PC02	ISLA total CD4 T cells	157	12	79019426	R	LOC105369863	Mixed	NA			B3
Chronic UD	PC02	ISLA total CD4 T cells	157	12	79019426	R	SYT1					
Chronic UD	PC02	ISLA total CD4 T cells	158	6	7194131	R	RREB1	Opposite	NA			A2
Chronic UD	PC02	ISLA total CD4 T cells	159	16	89677501	R	CDK10	Opposite	Name: AluSq2; Class: SINE; Family: Alu			A1
Chronic UD	PC02	ISLA total CD4 T cells	160	22	45738587	R	ATXN10	Opposite	NA			A1
Chronic UD	PC02	ISLA total CD4 T cells	161	6	90019258	R	BACH2	Same	NA	x		A2
Chronic UD	PC02	ISLA total CD4 T cells	162	6	90019258	R	BACH2	Same	NA	x		A2
Chronic UD	PC02	ISLA total CD4 T cells	163	13	77212905	F	MYCBP2	Opposite	NA			B3
Chronic UD	PC02	ISLA total CD4 T cells	164	17	82980000	F	B3GNTL1	Opposite	Name: L1MEc; Class: LINE; Family: L1			NA
Chronic UD	PC02	ISLA total CD4 T cells	165	15	41722463	R	MGA	Opposite	Name: L1ME3B; Class: LINE; Family: L1			A1
Chronic UD	PC02	ISLA total CD4 T cells	166	2	96199845	F	STARD7	Opposite	Name: L1MB7; Class: LINE; Family: L1			B1
Chronic UD	PC02	ISLA total CD4 T cells	167	17	2603716	R	PAFAH1B1	Opposite	NA			A1
Chronic UD	PC02	ISLA total CD4 T cells	168	19	14532336	R	TECR	Opposite	Name: AluSz; Class: SINE; Family: Alu			A1
Chronic UD	PC02	ISLA total CD4 T cells	169	14	72370041	R	RGS6	Opposite	NA			B1
Chronic UD	PC02	ISLA total CD4 T cells	170	1	41939754	F	HIVEP3	Opposite	Name: MIR3; Class: SINE; Family: MIR			A1
Chronic UD	PC02	ISLA total CD4 T cells	171	11	65870378	R	EFEMP2	Same	NA			A1
Chronic UD	PC02	ISLA total CD4 T cells	172	1	85099869	R	WDR63	Opposite	Name: L1PA4; Class: LINE; Family: L1			A2
Chronic UD	PC02	ISLA total CD4 T cells	173	15	90979650	F	PRC1-AS1	Mixed	NA			A1
Chronic UD	PC02	ISLA total CD4 T cells	173	15	90979650	F	PRC1					
Chronic UD	PC02	ISLA total CD4 T cells	174	19	25088581	F	Upstream: HAVCR1P1 (925.134 kb); Downstream: LOC105376906 (2431.523 kb)		Name: ALR/Alpha; Class: Satellite; Family: centr			NA
Chronic UD	PC02	ISLA total CD4 T cells	174	5	47489076	F	Upstream: HCN1 (1792.958 kb); Downstream: EMB (2907.116 kb)		Name: ALR/Alpha; Class: Satellite; Family: centr			NA
Chronic UD	PC02	ISLA total CD4 T cells	174	1	122683139	F	Upstream: EMBP1 (1111.251 kb); Downstream: LOC101929814 (20617.591 kb)		Name: ALR/Alpha; Class: Satellite; Family: centr			NA
Chronic UD	PC02	ISLA total CD4 T cells	175	10	73192737	R	FAM149B1	Mixed	NA			A1
Chronic UD	PC02	ISLA total CD4 T cells	175	10	73192737	R	LOC105378358					
Chronic UD	PC02	ISLA total CD4 T cells	176	17	78033109	R	TNRC6C	Opposite	NA			A1
Chronic UD	PC02	ISLA total CD4 T cells	177	17	4325256	F	UBE2G1	Opposite	Name: L1MC1; Class: LINE; Family: L1			A1
Chronic UD	PC02	ISLA total CD4 T cells	178	6	36221705	R	BRPF3	Opposite	NA			A1
Chronic UD	PC02	ISLA total CD4 T cells	179	X	42925673	F	LOC105373188	Same	Name: L1MA3; Class: LINE; Family: L1	x		NA
Chronic UD	PC02	ISLA total CD4 T cells	180	X	42925673	F	LOC105373188	Same	Name: L1MA3; Class: LINE; Family: L1	x		NA
Chronic UD	PC02	ISLA total CD4 T cells	181	X	42925673	F	LOC105373188	Same	Name: L1MA3; Class: LINE; Family: L1	x		NA
Chronic UD	PC02	ISLA total CD4 T cells	182	3	47218567	F	KIF9-AS1	Same	Name: LTR37A; Class: LTR; Family: ERV1			A1
Chronic UD	PC02	ISLA total CD4 T cells	183	12	100311768	R	SCYL2	Opposite	NA			B3
Chronic UD	PC02	ISLA total CD4 T cells	184	17	45301644	F	MAP3K14	Opposite	NA			A1
Chronic UD	PC02	ISLA total CD4 T cells	185	15	83036819	F	BTBD1	Opposite	Name: L1ME3; Class: LINE; Family: L1			NA
Chronic UD	PC02	ISLA total CD4 T cells	186	11	118264986	R	Upstream: MPZL2 (0.45 kb); Downstream: CD3E (39.594 kb)		Name: MIRc; Class: SINE; Family: MIR			A1
Chronic UD	PC02	ISLA total CD4 T cells	187	6	43198273	R	CUL9	Opposite	NA			A1
Chronic UD	PC02	ISLA total CD4 T cells	188	13	41816630	F	VWA8	Opposite	NA			A1
Chronic UD	PC02	ISLA total CD4 T cells	189	1	39417135	F	MACF1	Same	NA			A1
Chronic UD	PC02	ISLA total CD4 T cells	190	10	63575625	R	REEP3	Opposite	Name: HAL1; Class: LINE; Family: L1			B3
Chronic UD	PC02	ISLA total CD4 T cells	191	5	178612761	F	CLK4	Opposite	NA			B2
Chronic UD	PC02	ISLA total CD4 T cells	192	2	38575370	R	HNRNPLL	Same	NA			A2
Chronic UD	PC02	ISLA total CD4 T cells	193	2	70018498	F	PCBP1-AS1	Opposite	Name: L1MA8; Class: LINE; Family: L1			A1
Chronic UD	PC02	ISLA total CD4 T cells	194	17	41983090	R	DNAJC7	Same	NA			A1
Chronic UD	PC02	ISLA total CD4 T cells	195	3	37304803	R	GOLGA4	Opposite	NA			A2
Chronic UD	PC02	ISLA total CD4 T cells	196	6	149513687	F	PPIL4	Opposite	Name: L1ME4c; Class: LINE; Family: L1			A2
Chronic UD	PC02	ISLA total CD4 T cells	197	16	87338746	F	FBXO31	Opposite	NA			A1
Acute UD+5	PA02	ISLA total CD4 T cells	1	1	12398881	R	VPS13D	Opposite	Name: MIRb; Class: SINE; Family: MIR			A1
Acute UD+5	PA02	ISLA total CD4 T cells	2	17	42823093	F	BECN1	Opposite	Name: MIR; Class: SINE; Family: MIR			A1
Acute UD+5	PA02	ISLA total CD4 T cells	3	3	101770815	R	Upstream: CEP97 (0.253 kb); Downstream: NXPE3 (8.37 kb)			x		A2
Acute UD+5	PA02	ISLA total CD4 T cells	4	3	101770815	R	Upstream: CEP97 (0.253 kb); Downstream: NXPE3 (8.37 kb)			x		A2
Acute UD+5	PA02	ISLA total CD4 T cells	5	15	41735568	R	MGA	Opposite	Name: AluSc; Class: SINE; Family: Alu			A1
Acute UD+5	PA02	ISLA total CD4 T cells	6	1	120800693	F	LOC101929796	Same	Name: L1MB7; Class: LINE; Family: L1			NA
Acute UD+5	PA02	ISLA total CD4 T cells	6	1	146149670	R	Upstream: NBPF10 (4.866 kb); Downstream: NOTCH2NL (2.238 kb)		Name: L1MB7; Class: LINE; Family: L1			NA
Acute UD+5	PA02	ISLA total CD4 T cells	6	1	148600612	R	LOC100996763	Same	Name: L1MB7; Class: LINE; Family: L1			NA
Acute UD+5	PA02	ISLA total CD4 T cells	7	15	18470326	R	Upstream: N/A (0 kb); Downstream: LOC101059971 (1427.869 kb)		Name: ALR/Alpha; Class: Satellite; Family: centr			NA
Acute UD+5	PA02	ISLA total CD4 T cells	7	15	18472883	R	Upstream: N/A (0 kb); Downstream: LOC101059971 (1425.312 kb)		Name: ALR/Alpha; Class: Satellite; Family: centr			NA
Acute UD+5	PA02	ISLA total CD4 T cells	7	15	18484312	R	Upstream: N/A (0 kb); Downstream: LOC101059971 (1413.883 kb)		Name: ALR/Alpha; Class: Satellite; Family: centr			NA
Acute UD+5	PA02	ISLA total CD4 T cells	7	15	18529303	R	Upstream: N/A (0 kb); Downstream: LOC101059971 (1368.892 kb)		Name: ALR/Alpha; Class: Satellite; Family: centr			NA
Acute UD+5	PA02	ISLA total CD4 T cells	7	15	18616199	R	Upstream: N/A (0 kb); Downstream: LOC101059971 (1281.996 kb)		Name: ALR/Alpha; Class: Satellite; Family: centr			NA
Acute UD+5	PA02	ISLA total CD4 T cells	7	15	18736574	R	Upstream: N/A (0 kb); Downstream: LOC101059971 (1161.621 kb)		Name: ALR/Alpha; Class: Satellite; Family: centr			NA
Acute UD+5	PA02	ISLA total CD4 T cells	7	15	18759563	R	Upstream: N/A (0 kb); Downstream: LOC101059971 (1138.632 kb)		Name: ALR/Alpha; Class: Satellite; Family: centr			NA
Acute UD+5	PA02	ISLA total CD4 T cells	7	15	18764677	R	Upstream: N/A (0 kb); Downstream: LOC101059971 (1133.518 kb)		Name: ALR/Alpha; Class: Satellite; Family: centr			NA
Acute UD+5	PA02	ISLA total CD4 T cells	7	15	18797747	R	Upstream: N/A (0 kb); Downstream: LOC101059971 (1100.448 kb)		Name: ALR/Alpha; Class: Satellite; Family: centr			NA
Acute UD+5	PA02	ISLA total CD4 T cells	7	15	18814615	R	Upstream: N/A (0 kb); Downstream: LOC101059971 (1083.58 kb)		Name: ALR/Alpha; Class: Satellite; Family: centr			NA
Acute UD+5	PA02	ISLA total CD4 T cells	7	15	18891829	R	Upstream: N/A (0 kb); Downstream: LOC101059971 (1006.366 kb)		Name: ALR/Alpha; Class: Satellite; Family: centr			NA
Acute UD+5	PA02	ISLA total CD4 T cells	7	15	18985714	R	Upstream: N/A (0 kb); Downstream: LOC101059971 (912.481 kb)		Name: ALR/Alpha; Class: Satellite; Family: centr			NA
Acute UD+5	PA02	ISLA total CD4 T cells	7	15	19080992	R	Upstream: N/A (0 kb); Downstream: LOC101059971 (817.203 kb)		Name: ALR/Alpha; Class: Satellite; Family: centr			NA
Acute UD+5	PA02	ISLA total CD4 T cells	7	15	19160093	R	Upstream: N/A (0 kb); Downstream: LOC101059971 (738.102 kb)		Name: ALR/Alpha; Class: Satellite; Family: centr			NA
Acute UD+5	PA02	ISLA total CD4 T cells	7	15	19203043	R	Upstream: N/A (0 kb); Downstream: LOC101059971 (695.152 kb)		Name: ALR/Alpha; Class: Satellite; Family: centr			NA
Acute UD+5	PA02	ISLA total CD4 T cells	7	15	19218730	R	Upstream: N/A (0 kb); Downstream: LOC101059971 (679.465 kb)		Name: ALR/Alpha; Class: Satellite; Family: centr			NA
Acute UD+5	PA02	ISLA total CD4 T cells	7	15	19254158	R	Upstream: N/A (0 kb); Downstream: LOC101059971 (644.037 kb)		Name: ALR/Alpha; Class: Satellite; Family: centr			NA
Acute UD+5	PA02	ISLA total CD4 T cells	7	15	19531164	R	Upstream: N/A (0 kb); Downstream: LOC101059971 (367.031 kb)		Name: ALR/Alpha; Class: Satellite; Family: centr			NA
Acute UD+5	PA02	ISLA total CD4 T cells	8	11	128078665	R	Upstream: LOC387820 (137.01 kb); Downstream: LOC105369563 (88.533 kb)		Name: L1PA15-16; Class: LINE; Family: L1			B3
Acute UD+5	PA02	ISLA total CD4 T cells	9	1	235829443	F	LYST	Opposite	NA			A2
Acute UD+5	PA02	ISLA total CD4 T cells	10	19	46928320	R	ARHGAP35	Opposite	NA			B1
Acute UD+5	PA02	ISLA total CD4 T cells	11	13	49194351	R	FNDC3A	Opposite	NA			A2
Acute UD+5	PA02	ISLA total CD4 T cells	12	1	11135922	F	MTOR	Opposite	Name: AluSq2; Class: SINE; Family: Alu			A1
Acute UD+5	PA02	ISLA total CD4 T cells	13	1	24465107	F	NIPAL3	Same	Name: Tigger15a; Class: DNA; Family: TcMar-Tigger			A1
Acute UD+5	PA02	ISLA total CD4 T cells	14	11	3982408	R	STIM1	Opposite	Name: MIRb; Class: SINE; Family: MIR			A1
Acute UD+5	PA02	ISLA total CD4 T cells	15	10	35453449	F	CCNY	Same	Name: L1ME4a; Class: LINE; Family: L1			A2
Acute UD+5	PA02	ISLA total CD4 T cells	16	16	29585141	R	Upstream: SMG1P2 (18.922 kb); Downstream: LOC105371168 (12.65 kb)		Name: AluSq10; Class: SINE; Family: Alu			NA
Acute UD+5	PA02	ISLA total CD4 T cells	17	22	30183885	R	HORMAD2	Mixed	NA			A1
Acute UD+5	PA02	ISLA total CD4 T cells	17	22	30183885	R	LOC105372988		NA			
Acute UD+5	PA02	ISLA total CD4 T cells	18	19	42099047	R	POU2F2	Same	NA			A1
Acute UD+5	PA02	ISLA total CD4 T cells	19	22	39104175	R	Upstream: APOBEC3H (0.108 kb); Downstream: COX5BP7 (16.029 kb)		Name: AluSx; Class: SINE; Family: Alu			A1
Acute UD+5	PA02	ISLA total CD4 T cells	20	5	149524897	R	CSNK1A1	Same	NA			A2
Acute UD+5	PA02	ISLA total CD4 T cells	21	11	95973543	R	Upstream: RPL32P25 (9,919kb kb); Downstream: MAML2 (2.661 kb)		Name: MLT1K; Class: LTR; Family: ERVL-MaLR			A2
Acute UD+5	PA02	ISLA total CD4 T cells	22	3	67257643	R	Upstream: LOC105377145 (11.178 kb); Downstream: SUCLG2 (102.817 kb)		Name: L1MA9; Class: LINE; Family: L1			B3
Acute UD+5	PA02	ISLA total CD4 T cells	23	1	206218491	R	SRGAP2	Opposite	NA	x		A1
Acute UD+5	PA02	ISLA total CD4 T cells	24	1	206218491	R	SRGAP2	Opposite	NA	x		A1
Acute UD+5	PA02	ISLA total CD4 T cells	25	3	195316544	F	ACAP2	Opposite	Name: AluSz; Class: SINE; Family: Alu			A1
Acute UD+5	PA02	ISLA total CD4 T cells	26	16	29580030	R	SMG1P2	Same	NA			NA
Acute UD+5	PA02	ISLA total CD4 T cells	27	17	76792488	R	MFSD11	Opposite	Name: MIR; Class: SINE; Family: MIR			A1
Acute UD+5	PA02	ISLA total CD4 T cells	28	14	101812684	R	PPP2R5C	Opposite	NA			B1
Acute UD+5	PA02	ISLA total CD4 T cells	29	17	46375494	F	NSFP1	Same	Name: AluSq2; Class: SINE; Family: Alu			A1
Acute UD+5	PA02	ISLA total CD4 T cells	29	17	46593415	F	NSF	Same	Name: AluSq2; Class: SINE; Family: Alu			A1
Acute UD+5	PA02	ISLA total CD4 T cells	30	8	144302500	R	HSF1	Opposite	Name: AluSx4; Class: SINE; Family: Alu			A1
Acute UD+5	PA02	ISLA total CD4 T cells	31	19	52645279	R	ZNF83	Same	NA		x	B4
Acute UD+5	PA02	ISLA total CD4 T cells	32	16	72318187	R	LINC01572	Same	NA			B1
Acute UD+5	PA02	ISLA total CD4 T cells	33	2	218224127	R	ARPC2	Opposite	NA			B1
Acute UD+5	PA02	ISLA total CD4 T cells	34	17	16090310	R	NCOR1	Same	NA			A1
Acute UD+5	PA02	ISLA total CD4 T cells	35	8	144162786	R	MROH1	Opposite	Name: AluSx1; Class: SINE; Family: Alu			A1
Acute UD+5	PA02	ISLA total CD4 T cells	36	18	12344725	F	AFG3L2	Opposite	ame: (AATTA)n; Class: Simple_repeat; Family: Simple_repeat			A1
Acute UD+5	PA05	ISLA total CD4 T cells	37	4	54687778	F	KIT	Same	me: (ATACAC)n; Class: Simple_repeat; Family: Simple_rep	x		A2
Acute UD+5	PA05	ISLA total CD4 T cells	38	4	54687778	F	KIT	Same	me: (ATACAC)n; Class: Simple_repeat; Family: Simple_rep	x		A2
Acute UD+5	PA05	ISLA total CD4 T cells	39	1	146397561	R	Upstream: LOC100996756 (9.47 kb); Downstream: LOC105371235 (13.034 kb)		NA			NA
Acute UD+5	PA05	ISLA total CD4 T cells	40	14	100118723	R	EVL	Opposite	me: (CCTCA)n; Class: Simple_repeat; Family: Simple_repeat			B1
Acute UD+5	PA05	ISLA total CD4 T cells	41	12	133052038	R	ZNF84	Opposite	NA	x		A1
Acute UD+5	PA05	ISLA total CD4 T cells	42	12	133052038	R	ZNF84	Opposite	NA	x		A1
Acute UD+5	PA05	ISLA total CD4 T cells	43	12	133052038	R	ZNF84	Opposite	NA	x		A1
Acute UD+5	PA05	ISLA total CD4 T cells	44	22	13412480	F	Upstream: LOC105379428 (596.719 kb); Downstream: LOC100533637 (1912.751 kb)		Name: ALR/Alpha; Class: Satellite; Family: centr	x		NA
Acute UD+5	PA05	ISLA total CD4 T cells	44	21	11273970	F	Upstream: IGHV1OR21-1 (624.135 kb); Downstream: ANKRD30BP2 (1764.196 kb)		Name: ALR/Alpha; Class: Satellite; Family: centr			
Acute UD+5	PA05	ISLA total CD4 T cells	44	14	16531685	F	Upstream: LOC649620 (481.949 kb); Downstream: BNIP3P6 (1887.03 kb)		Name: ALR/Alpha; Class: Satellite; Family: centr			
Acute UD+5	PA05	ISLA total CD4 T cells	44	13	16409410	F	Upstream: N/A (0 kb); Downstream: LOC105379277 (1762.101 kb)		Name: ALR/Alpha; Class: Satellite; Family: centr			
Acute UD+5	PA05	ISLA total CD4 T cells	45	22	13412480	F	Upstream: LOC105379428 (596.719 kb); Downstream: LOC100533637 (1912.751 kb)		Name: ALR/Alpha; Class: Satellite; Family: centr	x		NA
Acute UD+5	PA05	ISLA total CD4 T cells	45	21	11273970	F	Upstream: IGHV1OR21-1 (624.135 kb); Downstream: ANKRD30BP2 (1764.196 kb)		Name: ALR/Alpha; Class: Satellite; Family: centr			
Acute UD+5	PA05	ISLA total CD4 T cells	45	14	16531685	F	Upstream: LOC649620 (481.949 kb); Downstream: BNIP3P6 (1887.03 kb)		Name: ALR/Alpha; Class: Satellite; Family: centr			
Acute UD+5	PA05	ISLA total CD4 T cells	45	13	16409410	F	Upstream: N/A (0 kb); Downstream: LOC105379277 (1762.101 kb)		Name: ALR/Alpha; Class: Satellite; Family: centr			
Acute UD+5	PA05	ISLA total CD4 T cells	46	3	197160904	R	DLG1	Same	NA			A1
Acute UD+5	PA05	ISLA total CD4 T cells	47	X	42924101	F	LOC105373188	Same	Name: L1MA3; Class: LINE; Family: L1			NA
Acute UD+5	PA05	ISLA total CD4 T cells	48	22	13800798	F	Upstream: LOC105379428 (985.037 kb); Downstream: LOC100533637 (1524.433 kb)		Name: ALR/Alpha; Class: Satellite; Family: centr	x		NA
Acute UD+5	PA05	ISLA total CD4 T cells	48	21	11662288	F	Upstream: IGHV1OR21-1 (1012.453 kb); Downstream: ANKRD30BP2 (1375.878 kb)		Name: ALR/Alpha; Class: Satellite; Family: centr			
Acute UD+5	PA05	ISLA total CD4 T cells	48	14	16920003	F	Upstream: LOC649620 (870.267 kb); Downstream: BNIP3P6 (1498.712 kb)		Name: ALR/Alpha; Class: Satellite; Family: centr			
Acute UD+5	PA05	ISLA total CD4 T cells	48	13	16797728	F	Upstream: N/A (0 kb); Downstream: LOC105379277 (1373.783 kb)		Name: ALR/Alpha; Class: Satellite; Family: centr			
Acute UD+5	PA05	ISLA total CD4 T cells	49	22	13800798	F	Upstream: LOC105379428 (985.037 kb); Downstream: LOC100533637 (1524.433 kb)		Name: ALR/Alpha; Class: Satellite; Family: centr	x		NA
Acute UD+5	PA05	ISLA total CD4 T cells	49	21	11662288	F	Upstream: IGHV1OR21-1 (1012.453 kb); Downstream: ANKRD30BP2 (1375.878 kb)		Name: ALR/Alpha; Class: Satellite; Family: centr			
Acute UD+5	PA05	ISLA total CD4 T cells	49	14	16920003	F	Upstream: LOC649620 (870.267 kb); Downstream: BNIP3P6 (1498.712 kb)		Name: ALR/Alpha; Class: Satellite; Family: centr			
Acute UD+5	PA05	ISLA total CD4 T cells	49	13	16797728	F	Upstream: N/A (0 kb); Downstream: LOC105379277 (1373.783 kb)		Name: ALR/Alpha; Class: Satellite; Family: centr			
Acute UD+5	PA05	ISLA total CD4 T cells	50	22	13800798	F	Upstream: LOC105379428 (985.037 kb); Downstream: LOC100533637 (1524.433 kb)		Name: ALR/Alpha; Class: Satellite; Family: centr			
Acute UD+5	PA05	ISLA total CD4 T cells	50	21	11662288	F	Upstream: IGHV1OR21-1 (1012.453 kb); Downstream: ANKRD30BP2 (1375.878 kb)		Name: ALR/Alpha; Class: Satellite; Family: centr	[illegible]		[illegible]

Acute UD+5	PA05	ISLA total CD4 T cells	50	14	16920003	F	Upstream: LOC649620 (870.267 kb); Downstream: BNIP3P6 (1498.712 kb)		Name: ALR/Alpha; Class: Satellite; Family: centr	x		NA
Acute UD+5	PA05	ISLA total CD4 T cells	50	13	16797728	F	Upstream: N/A (0 kb); Downstream: LOC105379277 (1373.783 kb)		Name: ALR/Alpha; Class: Satellite; Family: centr			
Acute UD+5	PA05	ISLA total CD4 T cells	51	6	31351170	F	Upstream: LOC105375015 (42.591 kb); Downstream: HLA-B (2.698 kb)		Name: MLT1N2; Class: LTR; Family: ERVL-MaLR			A1
Acute UD+5	PA05	ISLA total CD4 T cells	52	1	98878072	R	Upstream: SNX7 (117.572 kb); Downstream: LPPR5 (12.173 kb)		Name: L1PA6; Class: LINE; Family: L1			B3
Acute UD+5	PA05	ISLA total CD4 T cells	53	15	90455720	F	IQGAP1	Same	Name: MIRc; Class: SINE; Family: MIR			A1
Acute UD+5	PA05	ISLA total CD4 T cells	54	3	184935615	R	VPS8	Opposite	NA			A2
Acute UD+5	PA05	ISLA total CD4 T cells	55	9	25371592	F	Upstream: IZUMO3 (825.662 kb); Downstream: LOC105375995 (41.03 kb)		NA			B3
Acute UD+5	PA05	ISLA total CD4 T cells	56	20	2904098	F	PTPRA	Same	Name: AluJr; Class: SINE; Family: Alu			A1
Acute UD+5	PA15	ISLA total CD4 T cells	57	11	46559740	F	AMBRA1	Opposite	NA			A1
Acute UD+5	PA15	ISLA total CD4 T cells	58	9	35777507	F	NPR2	Same	Name: AluJr; Class: SINE; Family: Alu			A1
Acute UD+5	PA15	ISLA total CD4 T cells	59	6	33708412	F	UQCC2	Opposite	Name: MIRb; Class: SINE; Family: MIR			A1
Acute UD+5	PA15	ISLA total CD4 T cells	60	X	42853369	F	Upstream: PPP1R2P9 (75.134 kb); Downstream: LOC105373188 (67.02 kb)		NA			NA
Acute UD+5	PA15	ISLA total CD4 T cells	61	20	58987898	R	NELFCD	Opposite	NA			B2
Acute UD+5	PA15	ISLA total CD4 T cells	62	7	140142643	F	KDM7A	Opposite	Name: L1ME3B; Class: LINE; Family: L1			A2
Acute UD+5	PA15	ISLA total CD4 T cells	63	10	73456566	R	PPP3CB	Same	Name: L1MB8; Class: LINE; Family: L1			A1
Acute UD+5	PA19	ISLA total CD4 T cells	64	16	87375541	F	FBXO31	Opposite	NA			A1
Acute UD+5	PA19	ISLA total CD4 T cells	65	11	65423994	F	NEAT1	Same	NA	x		A1
Acute UD+5	PA19	ISLA total CD4 T cells	66	11	65423994	F	NEAT1	Same	NA	x		A1
Acute UD+5	PA19	ISLA total CD4 T cells	67	8	127552605	F	Upstream: CASC8 (70.466 kb); Downstream: LOC105375754 (111.947 kb)		NA			B3
Acute UD+5	PA19	ISLA total CD4 T cells	68	8	28129657	R	ELP3	Opposite	NA			A2
Acute UD+5	PA19	ISLA total CD4 T cells	69	17	44409846	R	GPATCH8	Same	NA			NA
Acute UD+5	PA19	ISLA total CD4 T cells	70	19	47109004	F	ZC3H4	Opposite	NA			A1
Acute UD+5	PA19	ISLA total CD4 T cells	71	17	81572735	F	NPLOC4	Opposite	NA			NA
Acute UD+5	PA19	ISLA total CD4 T cells	72	1	41845854	F	HIVEP3	Opposite	Name: AluSx1; Class: SINE; Family: Alu			A1
Acute UD+5	PA19	ISLA total CD4 T cells	73	2	38746323	F	SRSF7	Opposite	NA			A2
Acute UD+5	PA19	ISLA total CD4 T cells	74	14	68172739	R	RAD51B	Opposite	NA			A1
Acute UD+5	PA19	ISLA total CD4 T cells	75	17	39817412	F	IKZF3	Opposite	NA			A1
Acute UD+5	PA19	ISLA total CD4 T cells	76	1	145202262	F	LOC103091866 (uncharacterized)	Opposite	NA			B1
Acute UD+5	PA19	ISLA total CD4 T cells	76	1	145202262	F	LOC100996741	Opposite	NA			B1
Acute UD+5	PA19	ISLA total CD4 T cells	77	19	19431873	R	GATAD2A	Opposite	Name: L2b; Class: LINE; Family: L2			A1
Acute UD+5	PA19	ISLA total CD4 T cells	78	19	10170463	R	DNMT1	Same	Name: AluSq; Class: SINE; Family: Alu			B1
Acute UD+5	PA19	ISLA total CD4 T cells	79	6	31502742	R	MICB	Opposite	Name: L1MA3; Class: LINE; Family: L1			A1
Acute UD+5	PA19	ISLA total CD4 T cells	80	16	4061842	F	ADCY9	Opposite	Name: L1M4a2; Class: LINE; Family: L1			A1
Acute UD+5	PA19	ISLA total CD4 T cells	81	7	150463111	R	GIMAP8	Opposite	Name: L1M2; Class: LINE; Family: L1			A2
Acute UD+5	PA19	ISLA total CD4 T cells	82	9	36358517	F	RNF38	Opposite	NA			A1
Acute UD+5	PA19	ISLA total CD4 T cells	83	4	77048327	F	CCNI	Opposite	NA			A2
Acute UD+5	PA19	ISLA total CD4 T cells	84	6	36527661	F	STK38	Opposite	NA			A1
Acute UD+5	PA19	ISLA total CD4 T cells	85	19	35403187	R	Upstream: EEF1A1P7 (19.132 kb); Downstream: LINC01531 (2.42 kb)		Name: AluJr; Class: SINE; Family: Alu			B4
Acute UD+5	PA19	ISLA total CD4 T cells	86	9	121198469	F	RAB14	Mixed	NA			B3
Acute UD+5	PA19	ISLA total CD4 T cells	86	9	121198469	F	LOC105376337					
Acute UD+5	PA19	ISLA total CD4 T cells	87	17	78744725	F	CYTH1	Opposite	Name: Charlie24; Class: DNA; Family: hAT-Charlie			A1
Acute UD+5	PA19	ISLA total CD4 T cells	88	6	32965347	F	HLA-DM	Opposite	NA			A1
Acute UD+5	PA19	ISLA total CD4 T cells	89	5	141613123	F	DIAPH1	Opposite	NA			A2
Acute UD+5	PA19	ISLA total CD4 T cells	90	11	66121224	R	PACS1	Opposite	Name: Tigger1; Class: DNA; Family: TcMar-Tigger			A1
Acute UD+5	PA19	ISLA total CD4 T cells	91	19	52070743	F	ZNF841	Opposite	NA			B4
Acute UD+5	PA19	ISLA total CD4 T cells	92	1	55142258	F	USP24	Opposite	NA			A1
Acute UD+5	PA19	ISLA total CD4 T cells	93	1	146340500	R	LOC102724364	Same	NA			NA
Acute UD+5	PA19	ISLA total CD4 T cells	94	4	1945092	R	WHSC1	Opposite	NA			A1
Acute UD+5	PA19	ISLA total CD4 T cells	95	21	37428201	R	DYRK1A	Opposite	NA			A1
Acute UD+5	PA19	ISLA total CD4 T cells	96	17	75248058	R	GGA3	Same	NA			A1
Acute UD+5	PA19	ISLA total CD4 T cells	97	10	6478771	F	PRKCQ	Opposite	NA			A2
Acute UD+5	PA19	ISLA total CD4 T cells	98	17	43515824	R	DHX8	Opposite	NA			A1
Acute UD+5	PA35	ISLA total CD4 T cells	99	15	63552785	R	USP3	Opposite	NA			A1
Acute UD+5	PA35	ISLA total CD4 T cells	100	7	150610748	F	Upstream: EIF2AP2 (6.1 kb); Downstream: LOC100288724 (3.322 kb)		Name: MER57A-int; Class: LTR; Family: ERV1			A2
Acute UD+5	PA35	ISLA total CD4 T cells	101	15	85492020	F	AKAP13	Same	NA			A1
Acute UD+5	PA35	ISLA total CD4 T cells	102	15	21744233	R	Upstream: LOC102724971 (1.335 kb); Downstream: LOC105379208 (8.515 kb)		NA			NA
Acute UD+5	PA35	ISLA total CD4 T cells	103	15	81291035	R	IL16	Opposite	Name: MIR3; Class: SINE; Family: MIR			B1
Acute UD+5	PA35	ISLA total CD4 T cells	104	1	198617256	R	Upstream: ATP6V1G3 (76.311 kb); Downstream: PTPRC (21.712 kb)		Name: L1ME3A; Class: LINE; Family: L1			A2
Acute UD+5	PA35	ISLA total CD4 T cells	105	11	66167895	F	PACS1	Same	Name: L1ME3F; Class: LINE; Family: L1			A1
Acute UD+5	PA35	ISLA total CD4 T cells	106	2	203745878	R	Upstream: CD28 (6.966 kb); Downstream: KRT18P39 (18.355 kb)		Name: MLT1L; Class: LTR; Family: ERVL-MaLR			A2
Acute UD+5	PA35	ISLA total CD4 T cells	107	12	91978333	F	Upstream: LOC105369901 (17.235 kb); Downstream: C12orf79 (6.643 kb)		Name: MLT1C; Class: LTR; Family: ERVL-MaLR			B3
Acute UD+5	PA35	ISLA total CD4 T cells	108	18	18548151	R	Upstream: LOC644669 (3222.232 kb); Downstream: ROCK1 (2401.589 kb)		Name: ALR/Alpha; Class: Satellite; Family: centr			B2
Acute UD+5	PA35	ISLA total CD4 T cells	109	Unknown	58877	F	LOC283788	Opposite	NA			NA
Acute UD+5	PA35	ISLA total CD4 T cells	110	9	127966317	F	FAM102A	Opposite	NA			A1
Acute UD+5	PA35	ISLA total CD4 T cells	111	13	91134914	R	LINC00379	Mixed	NA			B3
Acute UD+5	PA35	ISLA total CD4 T cells	111	13	91134914	R	LOC105370312					
Acute UD+5	PA35	ISLA total CD4 T cells	112	9	111385601	F	KIAA0368 (other name: ECPAS)	Opposite	Name: L2a; Class: LINE; Family: L2			B3
Acute UD+5	PA35	ISLA total CD4 T cells	113	13	74472254	R	Upstream: LINC00381 (37.095 kb); Downstream: LINC00347 (80.589 kb)		NA			A2
Acute UD+5	PA35	ISLA total CD4 T cells	114	11	7411651	R	SYT9	Opposite	Name: L1MA4A; Class: LINE; Family: L1			B1
Acute UD+5	PA35	ISLA total CD4 T cells	115	9	121182216	F	RAB14	Opposite	NA			B3
Acute UD+5	PA35	ISLA total CD4 T cells	116	5	180231324	F	Upstream: RPS8P7 (4.324 kb); Downstream: MAPK9 (2.27 kb)		NA			A1
Acute UD+5	PA35	ISLA total CD4 T cells	117	20	27199894	R	Upstream: MIR663AHG (990.661 kb); Downstream: LOC100289097 (1357.196 kb)		Name: ALR/Alpha; Class: Satellite; Family: centr			NA
Acute UD+5	PA35	ISLA total CD4 T cells	118	16	89432161	F	ANKRD11	Mixed	NA			A1
Acute UD+5	PA35	ISLA total CD4 T cells	118	16	89432161	F	LOC101927817					
Acute UD+5	PA35	ISLA total CD4 T cells	119	18	46092975	F	ATP5A1	Opposite	Name: AluSz6; Class: SINE; Family: Alu			B1
Acute UD+5	PA35	ISLA total CD4 T cells	120	5	52762119	F	Upstream: LOC105378964 (10.343 kb); Downstream: PELO (25.821 kb)		Name: MLT1A; Class: LTR; Family: ERVL-MaLR			B3
Acute UD+5	PA35	ISLA total CD4 T cells	121	14	52469896	F	TXNDC16	Opposite	Name: L1ME4a; Class: LINE; Family: L1			A2
Acute UD+5	PA35	ISLA total CD4 T cells	122	Y	56684982	R	Upstream: PARP4P1 (30050.327 kb); Downstream: CTBP2P1 (170.261 kb)		Name: (AATGGAATGG)n; Class: Simple_repeat; Family: Simple_repeat			NA
Acute UD+5	PA35	ISLA total CD4 T cells	123	3	46535876	F	LRRC2	Opposite	Name: MIR; Class: SINE; Family: MIR			A1
Acute UD+5	PA35	ISLA total CD4 T cells	124	22	14033896	R	Upstream: LOC105379428 (1218.135 kb); Downstream: LOC100533637 (1291.335 kb)		Name: ALR/Alpha; Class: Satellite; Family: centr			NA
Acute UD+5	PA35	ISLA total CD4 T cells	124	21	11895386	R	Upstream: IGHV1OR21-1 (1245.551 kb); Downstream: ANKRD30BP2 (1142.78 kb)		Name: ALR/Alpha; Class: Satellite; Family: centr			NA
Acute UD+5	PA35	ISLA total CD4 T cells	124	14	17153101	R	Upstream: LOC649620 (1103.365 kb); Downstream: BNIP3P6 (1265.614 kb)		Name: ALR/Alpha; Class: Satellite; Family: centr			NA
Acute UD+5	PA35	ISLA total CD4 T cells	124	13	17030826	R	Upstream: N/A (0 kb); Downstream: LOC105379277 (1140.685 kb)		Name: ALR/Alpha; Class: Satellite; Family: centr			NA
Acute UD	PA02	ISLA total CD4 T cells	1	2	101279929	R	RNF149	Same	Name: L1MD; Class: LINE; Family: L1			B1
Acute UD	PA02	ISLA total CD4 T cells	2	22	41478753	R	ACO2	Opposite	NA			A1
Acute UD	PA02	ISLA total CD4 T cells	2	22	41478753	R	LOC105369160	Opposite	NA			A1
Acute UD	PA02	ISLA total CD4 T cells	3	19	1125748	F	SBNO2	Opposite	Name: AluJb; Class: SINE; Family: Alu			A1
Acute UD	PA02	ISLA total CD4 T cells	4	14	91817061	R	TC2N	Same	Name: L1MC5; Class: LINE; Family: L1			A1
Acute UD	PA02	ISLA total CD4 T cells	5	1	10128745	F	UBE4B	Same	NA			A1
Acute UD	PA02	ISLA total CD4 T cells	6	19	14455293	R	PKN1	Opposite	Name: AluJb; Class: SINE; Family: Alu			A1
Acute UD	PA02	ISLA total CD4 T cells	7	11	66631008	F	RBM14-RBM4	Same	Name: AluSq2; Class: SINE; Family: Alu			A1
Acute UD	PA02	ISLA total CD4 T cells	8	20	50745345	R	PARD6B	Opposite	Name: AluSx; Class: SINE; Family: Alu			B1
Acute UD	PA02	ISLA total CD4 T cells	9	3	51462107	F	VPRBP	Opposite	NA			A1
Acute UD	PA02	ISLA total CD4 T cells	10	19	39967224	R	Upstream: PRR13P5 (23.472 kb); Downstream: PSMC4 (3.781 kb)		NA			A1
Acute UD	PA02	ISLA total CD4 T cells	11	17	7877521	R	NAA38	Same	Name: L1ME3Cz; Class: LINE; Family: L1			A1
Acute UD	PA02	ISLA total CD4 T cells	12	6	90054089	R	BACH2	Same	Name: L1PA4; Class: LINE; Family: L1			A2
Acute UD	PA02	ISLA total CD4 T cells	13	4	2875768	F	ADD1	Same	NA			A1
Acute UD	PA02	ISLA total CD4 T cells	14	16	53844540	F	FTO	Same	Name: AluSq; Class: SINE; Family: Alu			A2
Acute UD	PA02	ISLA total CD4 T cells	15	13	98463475	F	STK24	Opposite	NA			B3
Acute UD	PA02	ISLA total CD4 T cells	16	11	121055444	R	TBCEL	Opposite	NA			A2
Acute UD	PA02	ISLA total CD4 T cells	17	14	69118646	F	DCAF5	Opposite	NA			A1
Acute UD	PA02	ISLA total CD4 T cells	18	16	89475524	F	ANKRD11	Opposite	NA			A1
Acute UD	PA02	ISLA total CD4 T cells	19	3	157110074	R	LINC00880	Same	NA			B3
Acute UD	PA02	ISLA total CD4 T cells	20	9	129959328	F	FNBP1	Opposite	Name: AluSx; Class: SINE; Family: Alu			A1
Acute UD	PA02	ISLA total CD4 T cells	21	7	11704014	R	THSD7A	Same	NA			B3
Acute UD	PA02	ISLA total CD4 T cells	22	16	29430071	R	SMG1P6	Same	NA			NA
Acute UD	PA02	ISLA total CD4 T cells	23	1	113767261	F	RSBN1	Opposite	NA			B1
Acute UD	PA02	ISLA total CD4 T cells	24	14	68223796	R	RAD51B	Opposite	NA			A1
Acute UD	PA02	ISLA total CD4 T cells	25	17	18554106	R	CCDC144B	Same	NA			B1
Acute UD	PA02	ISLA total CD4 T cells	26	X	13034253	F	Upstream: TMSB4X (57.026 kb); Downstream: FAM9C (1.364 kb)		Name: L1MC1; Class: LINE; Family: L1			NA
Acute UD	PA02	ISLA total CD4 T cells	27	21	5323595	F	Upstream: LOC102724200 (158.131 kb); Downstream: LOC105379487 (175.788 kb)		Name: ALR/Alpha; Class: Satellite; Family: centr			NA
Acute UD	PA02	ISLA total CD4 T cells	27	21	7253616	F	Upstream: LOC102724931 (122.693 kb); Downstream: LOC105379502 (130.357 kb)		Name: ALR/Alpha; Class: Satellite; Family: centr			NA
Acute UD	PA02	ISLA total CD4 T cells	28	17	8906550	F	PIK3R5	Opposite	NA			B1
Acute UD	PA02	ISLA total CD4 T cells	29	11	18473861	R	LDHAL6A	Opposite	Name: L1M5; Class: LINE; Family: L1			A1
Acute UD	PA02	ISLA total CD4 T cells	30	6	90768860	R	Upstream: LOC105377893 (126.176 kb); Downstream: MIR4643 (752.8 kb)		NA			B3
Acute UD	PA02	ISLA total CD4 T cells	31	6	32960442	R	Upstream: HLA-DMA (7.32 kb); Downstream: LOC105375109 (6.263 kb)		NA			A1
Acute UD	PA02	ISLA total CD4 T cells	32	15	65170417	F	CLPX	Opposite	NA			A1
Acute UD	PA02	ISLA total CD4 T cells	33	4	24558912	R	DHX15	Same	Name: MIR3; Class: SINE; Family: MIR			A2
Acute UD	PA02	ISLA total CD4 T cells	34	9	128017133	R	Upstream: FAM102A (36.106 kb); Downstream: NAIF1 (44.1 kb)		Name: L1MC3; Class: LINE; Family: L1			A1
Acute UD	PA02	ISLA total CD4 T cells	35	12	47508613	R	Upstream: LOC100127978 (22.891 kb); Downstream: RPAP3 (153.319 kb)		Name: L1PA4; Class: LINE; Family: L1			B1
Acute UD	PA02	ISLA total CD4 T cells	36	12	8063233	F	C3AR1	Opposite	Name: AluSc8; Class: SINE; Family: Alu			A1
Acute UD	PA02	ISLA total CD4 T cells	37	15	72549997	F	ARIH1	Same	NA			A1
Acute UD	PA02	ISLA total CD4 T cells	38	1	206745816	F	Upstream: MAPKAPK2 (11.531 kb); Downstream: LOC105372877 (2.515 kb)		NA			A1
Acute UD	PA02	ISLA total CD4 T cells	39	18	46253090	F	C18orf25	Same	NA			A1
Acute UD	PA02	ISLA total CD4 T cells	40	4	2698692	F	FAM193A	Same	NA			A1
Acute UD	PA02	ISLA total CD4 T cells	41	1	33425147	F	PHC2	Opposite	NA			A1
Acute UD	PA02	ISLA total CD4 T cells	42	8	6723765	F	AGPAT5	Same	NA			B2
Acute UD	PA02	ISLA total CD4 T cells	43	17	80359352	F	RNF213	Mixed	Name: AluJr; Class: SINE; Family: Alu			A1
Acute UD	PA02	ISLA total CD4 T cells	43	17	80359352	F	LOC100294362					
Acute UD	PA02	ISLA total CD4 T cells	44	12	68640723	F	RAP1B	Same	NA			A2
Acute UD	PA02	ISLA total CD4 T cells	45	17	39522704	R	CDK12	Opposite	Name: AluSx1; Class: SINE; Family: Alu			B1
Acute UD	PA02	ISLA total CD4 T cells	46	19	11476733	R	ELAVL3	Same	Name: AluSx1; Class: SINE; Family: Alu			A1
Acute UD	PA02	ISLA total CD4 T cells	47	7	8421546	R	Upstream: LOC100505938 (78.265 kb); Downstream: NXPH1 (12.409 kb)		NA			B3
Acute UD	PA02	ISLA total CD4 T cells	48	X	40072600	R	BCOR	Same	NA			
Acute UD	PA02	ISLA total CD4 T cells	49	6	35652352	F	FKBP5	Mixed	Name: MER11C; Class: LTR; Family: ERVK			A1
Acute UD	PA02	ISLA total CD4 T cells	49	6	35652352	F	LOC105375033					
Acute UD	PA02	ISLA total CD4 T cells	50	1	154239715	R	UBAP2L	Opposite	Name: L3; Class: LINE; Family: CR1			A1
Acute UD	PA02	ISLA total CD4 T cells	51	1	145947597	F	LIX1L	Mixed	NA			NA
Acute UD	PA02	ISLA total CD4 T cells	51	1	145947597	F	LOC105371261		NA			
Acute UD	PA02	ISLA total CD4 T cells	52	3	197220046	R	DLG1	Same	NA			A1
Acute UD	PA02	ISLA total CD4 T cells	53	11	66088352	F	PACS1	Same	Name: L1ME3A; Class: LINE; Family: L1			A1
Acute UD	PA02	ISLA total CD4 T cells	54	1	156290411	R	TMEM79	Opposite	Name: FLAM_C; Class: SINE; Family: Alu			A1
Acute UD	PA02	ISLA total CD4 T cells	55	11	96288113	R	MAML2	Same	NA			B3
Acute UD	PA02	ISLA total CD4 T cells	56	2	87163949	F	LOC102724642	Same	Name: MER47B; Class: DNA; Family: TcMar-Tigger			NA
Acute UD	PA02	ISLA total CD4 T cells	57	15	74772126	F	Upstream: CYP1A2 (15.526 kb); Downstream: CSK (9.958 kb)		NA			A1

Acute UD	PA02	ISLA total CD4 T cells	58	11	67178765	R	KDM2A	Opposite	Name: L1MB7; Class: LINE; Family: L1			A1
Acute UD	PA02	ISLA total CD4 T cells	59	4	152014907	F	Upstream: RNA5SP169 (43.294 kb); Downstream: LOC100996286 (85.846 kb)		Name: MIRc; Class: SINE; Family: MIR			A2
Acute UD	PA02	ISLA total CD4 T cells	60	17	40250378	R	WIPF2	Opposite	Name: AluY; Class: SINE; Family: Alu			A1
Acute UD	PA02	ISLA total CD4 T cells	61	9	109187095	F	EPB41L4B	Opposite	NA			B3
Acute UD	PA02	ISLA total CD4 T cells	62	20	63732284	R	ZGPAT	Opposite	Name: (GT)n; Class: Simple_repeat; Family: Simple_repeat			NA
Acute UD	PA02	ISLA total CD4 T cells	63	3	33057396	F	GLB1	Opposite	NA			A2
Acute UD	PA02	ISLA total CD4 T cells	64	14	101764727	R	PPP2R5C	Opposite	NA			B1
Acute UD	PA02	ISLA total CD4 T cells	65	12	123339203	R	SBNO1	Same	NA			A1
Acute UD	PA02	ISLA total CD4 T cells	66	17	64101072	F	ERN1	Opposite	NA			B1
Acute UD	PA02	ISLA total CD4 T cells	67	11	66093386	F	PACS1	Same	Name: L1PA3; Class: LINE; Family: L1			A1
Acute UD	PA02	ISLA total CD4 T cells	68	17	35910289	R	LRRC37A8P	Same	NA			A1
Acute UD	PA02	ISLA total CD4 T cells	69	19	19404129	R	GATAD2A	Opposite	NA			A1
Acute UD	PA02	ISLA total CD4 T cells	70	1	36027319	R	AGO3	Opposite	NA			A1
Acute UD	PA02	ISLA total CD4 T cells	71	17	80733722	R	RPTOR	Opposite	NA			A1
Acute UD	PA02	ISLA total CD4 T cells	72	7	105823631	F	ATXN7L1	Opposite	NA			A2
Acute UD	PA02	ISLA total CD4 T cells	73	14	91009218	R	RPS6KA5	Same	Name: MLT1A0; Class: LTR; Family: ERVL-MaLR			B1
Acute UD	PA02	ISLA total CD4 T cells	74	1	39430826	F	MACF1	Same	NA			A1
Acute UD	PA02	ISLA total CD4 T cells	75	17	7486219	F	POLR2A	Same	Name: L2a; Class: LINE; Family: L2			A1
Acute UD	PA02	ISLA total CD4 T cells	76	17	76168668	R	RNF157	Same	Name: HAL1; Class: LINE; Family: L1			A1
Acute UD	PA02	ISLA total CD4 T cells	77	1	193052024	F	UCHL5	Opposite	NA			A2
Acute UD	PA05	ISLA total CD4 T cells	78	14	65484270	R	FUT8	Opposite	Name: L1MEc; Class: LINE; Family: L1			A2
Acute UD	PA05	ISLA total CD4 T cells	79	19	34991660	R	Upstream: ZNF792 (27.378 kb); Downstream: GRAMD1A (3.124 kb)		NA			B4
Acute UD	PA05	ISLA total CD4 T cells	80	9	60664872	F	Upstream: LOC102724904 (17511.142 kb); Downstream: LOC105379434 (233.442 kb)		NA			NA
Acute UD	PA05	ISLA total CD4 T cells	81	8	21965510	F	XPO7	Same	NA			A1
Acute UD	PA05	ISLA total CD4 T cells	82	14	106466153	R	IGH	Same	NA			NA
Acute UD	PA05	ISLA total CD4 T cells	83	4	53411784	F	FIP1L1	Same	NA			A2
Acute UD	PA05	ISLA total CD4 T cells	84	10	74367716	R	ADK	Opposite	Name: Tigger17c; Class: DNA; Family: TcMar-Tigger			A1
Acute UD	PA05	ISLA total CD4 T cells	85	2	112317276	R	ZC3H6	Opposite	NA			B1
Acute UD	PA05	ISLA total CD4 T cells	86	X	136717346	F	ARHGEF6	Opposite	Name: L1MCa; Class: LINE; Family: L1			NA
Acute UD	PA05	ISLA total CD4 T cells	87	6	26278586	R	Upstream: HIST1H2BI (5.174 kb); Downstream: LOC105374985 (1.768 kb)		NA			A1
Acute UD	PA05	ISLA total CD4 T cells	88	1	143213647	R	Upstream: EMBP1 (21641.759 kb); Downstream: LOC101929814 (87.083 kb)		NA			NA
Acute UD	PA05	ISLA total CD4 T cells	88	1	143215890	R	Upstream: EMBP1 (21644.002 kb); Downstream: LOC101929814 (84.84 kb)		NA			NA
Acute UD	PA05	ISLA total CD4 T cells	89	5	143390051	F	NR3C1	Opposite	NA			B3
Acute UD	PA05	ISLA total CD4 T cells	90	19	57215181	R	ZNF264	Opposite	Name: L1MDa; Class: LINE; Family: L1		x	B2
Acute UD	PA05	ISLA total CD4 T cells	91	1	116552735	R	CD58	Same	NA			B1
Acute UD	PA05	ISLA total CD4 T cells	92	16	28172454	R	XPO6	Same	NA			A1
Acute UD	PA05	ISLA total CD4 T cells	93	15	78039654	R	TBC1D2B	Same	NA			B1
Acute UD	PA14	ISLA total CD4 T cells	94	12	122893619	R	VPS37B	Same	NA			A1
Acute UD	PA14	ISLA total CD4 T cells	95	19	17816256	F	Upstream: B3GNT3 (2.68 kb); Downstream: INSL3 (0.257 kb)		NA			A1
Acute UD	PA14	ISLA total CD4 T cells	96	14	21227575	F	HNRNPC	Opposite	Name: MADE1; Class: DNA; Family: TcMar-Mariner			B1
Acute UD	PA14	ISLA total CD4 T cells	97	1	8483878	F	RERE	Opposite	NA			A1
Acute UD	PA14	ISLA total CD4 T cells	98	6	25418367	R	LRRC16A	Opposite	Name: MER45A; Class: DNA; Family: hAT-Tip100			A2
Acute UD	PA14	ISLA total CD4 T cells	99	12	7111727	R	C1RL-AS1	Opposite	Name: L1MEf; Class: LINE; Family: L1			A1
Acute UD	PA14	ISLA total CD4 T cells	100	16	31186808	R	FUS	Opposite	NA			A1
Acute UD	PA14	ISLA total CD4 T cells	101	17	32194163	F	RHOT1	Same	Name: AluJo; Class: SINE; Family: Alu			B1
Acute UD	PA14	ISLA total CD4 T cells	102	12	48077018	R	SENP1	Same	Name: L1PA16; Class: LINE; Family: L1			A2
Acute UD	PA14	ISLA total CD4 T cells	103	14	64112180	R	SYNE2	Opposite	NA			A2
Acute UD	PA14	ISLA total CD4 T cells	104	16	89763735	F	FANCA	Opposite	Name: AluSg; Class: SINE; Family: Alu			A1
Acute UD	PA14	ISLA total CD4 T cells	105	7	150466034	R	GIMAP8	Opposite	Name: MIRc; Class: SINE; Family: MIR			A2
Acute UD	PA14	ISLA total CD4 T cells	106	1	89640800	R	LRRC8C	Opposite	NA			A2
Acute UD	PA14	ISLA total CD4 T cells	107	X	97192689	R	DIAPH2	Opposite	Name: Charlie18a; Class: DNA; Family: hAT-Charlie			NA
Acute UD	PA14	ISLA total CD4 T cells	108	21	9110910	F	TEKT4P2	Opposite	Name: L1MB2; Class: LINE; Family: L1			NA
Acute UD	PA14	ISLA total CD4 T cells	109	11	58584204	R	ZFP91	Opposite	NA			B2
Acute UD	PA14	ISLA total CD4 T cells	109	11	58584204	R	ZFP91-CNTF	Opposite	NA			B2
Acute UD	PA14	ISLA total CD4 T cells	110	19	8462523	R	HNRNPM	Opposite	NA			A1
Acute UD	PA14	ISLA total CD4 T cells	111	1	44700254	R	C1orf228	Mixed	Name: MIRc; Class: SINE; Family: MIR			A1
Acute UD	PA14	ISLA total CD4 T cells	111	1	44700254	R	LOC105378690					
Acute UD	PA14	ISLA total CD4 T cells	112	16	29581831	F	Upstream: SMG1P2 (15.612 kb); Downstream: LOC105371168 (15.96 kb)		NA			NA
Acute UD	PA14	ISLA total CD4 T cells	113	6	35633996	R	FKBP5	Mixed	NA			A1
Acute UD	PA14	ISLA total CD4 T cells	113	6	35633996	R	LOC105375032		NA			
Acute UD	PA14	ISLA total CD4 T cells	114	19	19352648	F	MAU2	Same	Name: MIRc; Class: SINE; Family: MIR			A1
Acute UD	PA14	ISLA total CD4 T cells	115	16	29743045	R	C16orf54	Same	Name: AluJo; Class: SINE; Family: Alu			A1
Acute UD	PA14	ISLA total CD4 T cells	116	17	4689706	F	PELP1	Opposite	Name: L1PB1; Class: LINE; Family: L1			A1
Acute UD	PA14	ISLA total CD4 T cells	117	19	39828609	F	DYRK1B	Opposite	NA			A1
Acute UD	PA14	ISLA total CD4 T cells	118	9	114357504	R	AKNA	Same	NA			A2
Acute UD	PA14	ISLA total CD4 T cells	119	4	2678777	R	FAM193A	Opposite	Name: L1MB3; Class: LINE; Family: L1			A1
Acute UD	PA14	ISLA total CD4 T cells	120	17	78392778	R	PGS1	Opposite	NA			A1
Acute UD	PA14	ISLA total CD4 T cells	121	2	38598935	R	HNRNPLL	Same	NA			A2
Acute UD	PA14	ISLA total CD4 T cells	122	1	8635699	R	RERE	Same	NA			A1
Acute UD	PA14	ISLA total CD4 T cells	123	6	31808834	F	Upstream: LSM2 (1.85 kb); Downstream: HSPA1L (0.785 kb)		Name: AluSq2; Class: SINE; Family: Alu			A1
Acute UD	PA14	ISLA total CD4 T cells	124	17	83086370	R	METRNL	Opposite	NA			NA
Acute UD	PA14	ISLA total CD4 T cells	125	16	89985153	R	AFG3L1P	Opposite	Name: AluSz; Class: SINE; Family: Alu			A1
Acute UD	PA14	ISLA total CD4 T cells	126	11	128560479	R	ETS1	Same	Name: MIRb; Class: SINE; Family: MIR			A2
Acute UD	PA14	ISLA total CD4 T cells	127	3	52181126	F	Upstream: POC1A (26.436 kb); Downstream: ALDOAP1 (11.919 kb)		Name: L1P3; Class: LINE; Family: L1			A1
Acute UD	PA14	ISLA total CD4 T cells	128	19	9996791	F	COL5A3	Opposite	me: GA-rich; Class: Low_complexity; Family: Low_complexity			B4
Acute UD	PA14	ISLA total CD4 T cells	129	9	137127100	F	Upstream: DPP7 (9.12 kb); Downstream: GRIN1 (12.012 kb)		NA			A1
Acute UD	PA14	ISLA total CD4 T cells	130	3	71062589	F	FOXP1	Opposite	NA			B3
Acute UD	PA14	ISLA total CD4 T cells	131	11	247389	R	PSMD13	Opposite	NA			A1
Acute UD	PA14	ISLA total CD4 T cells	132	6	31746608	R	MSH5-SAPCD1	Opposite	Name: AluSz; Class: SINE; Family: Alu			A1
Acute UD	PA14	ISLA total CD4 T cells	132	6	31746608	R	MSH5	Opposite	Name: AluSz; Class: SINE; Family: Alu			A1
Acute UD	PA14	ISLA total CD4 T cells	133	16	597969	F	RAB40C	Same	Name: AluSc; Class: SINE; Family: Alu			A1
Acute UD	PA14	ISLA total CD4 T cells	134	3	196192057	F	Upstream: LINC00885 (31.167 kb); Downstream: LOC105374303 (0.728 kb)		NA			A1
Acute UD	PA14	ISLA total CD4 T cells	135	3	48949677	R	ARIH2	Opposite	Name: L1ME1; Class: LINE; Family: L1			A1
Acute UD	PA14	ISLA total CD4 T cells	136	4	122731518	F	BBS12	Mixed	NA			A2
Acute UD	PA14	ISLA total CD4 T cells	136	4	122731518	F	CETN4P		NA			
Acute UD	PA14	ISLA total CD4 T cells	137	22	50374545	R	PPP6R2	Opposite	Name: L1MEd; Class: LINE; Family: L1			A1
Acute UD	PA14	ISLA total CD4 T cells	138	5	177548188	R	FAM193B	Same	Name: L2c; Class: LINE; Family: L2			A1
Acute UD	PA14	ISLA total CD4 T cells	139	12	122512892	R	RSRC2	Same	NA			A1
Acute UD	PA14	ISLA total CD4 T cells	140	16	75651864	R	TERF2IP	Opposite	NA			A2
Acute UD	PA14	ISLA total CD4 T cells	141	17	44181724	F	ASB16-AS1	Opposite	Name: AluSx; Class: SINE; Family: Alu	x		A1
Acute UD	PA14	ISLA total CD4 T cells	142	17	44181724	F	ASB16-AS1	Opposite	Name: AluSx; Class: SINE; Family: Alu	x		A1
Acute UD	PA14	ISLA total CD4 T cells	143	15	61925645	F	VPS13C	Opposite	NA			B1
Acute UD	PA14	ISLA total CD4 T cells	144	16	21471606	F	SMG1P3	Opposite	NA			NA
Acute UD	PA14	ISLA total CD4 T cells	145	2	27633306	R	GPN1	Opposite	NA	x		A1
Acute UD	PA14	ISLA total CD4 T cells	146	2	27633306	R	GPN1	Opposite	NA	x		A1
Acute UD	PA14	ISLA total CD4 T cells	147	2	27633306	R	GPN1	Opposite	NA	x		A1
Acute UD	PA14	ISLA total CD4 T cells	148	2	27633306	R	GPN1	Opposite	NA	x		A1
Acute UD	PA14	ISLA total CD4 T cells	149	2	27633306	R	GPN1	Opposite	NA	x		A1
Acute UD	PA14	ISLA total CD4 T cells	150	2	27633306	R	GPN1	Opposite	NA	x		A1
Acute UD	PA14	ISLA total CD4 T cells	151	2	27633306	R	GPN1	Opposite	NA	x		A1
Acute UD	PA14	ISLA total CD4 T cells	152	2	27633306	R	GPN1	Opposite	NA	x		A1
Acute UD	PA14	ISLA total CD4 T cells	153	2	27633306	R	GPN1	Opposite	NA	x		A1
Acute UD	PA14	ISLA total CD4 T cells	154	3	142137523	F	TFDP2	Opposite	Name: L1PA6; Class: LINE; Family: L1			A2
Acute UD	PA14	ISLA total CD4 T cells	155	6	33654799	R	ITPR3	Opposite	Name: L3; Class: LINE; Family: CR1			A1
Acute UD	PA14	ISLA total CD4 T cells	156	19	13170477	R	Upstream: RPL12P42 (11.603 kb); Downstream: CACNA1A (35.965 kb)		Name: AluJr; Class: SINE; Family: Alu			A1
Acute UD	PA14	ISLA total CD4 T cells	157	4	938043	F	TMEM175	Same	Name: L1ME1; Class: LINE; Family: L1			A1
Acute UD	PA14	ISLA total CD4 T cells	158	6	43533149	R	POLR1C	Mixed	Name: AluSz; Class: SINE; Family: Alu			A1
Acute UD	PA14	ISLA total CD4 T cells	158	6	43533149	R	XPO5					
Acute UD	PA14	ISLA total CD4 T cells	159	17	4871953	R	MINK1	Opposite	Name: AluJo; Class: SINE; Family: Alu			A1
Acute UD	PA14	ISLA total CD4 T cells	160	19	1272838	F	CIRBP	Same	NA			A1
Acute UD	PA14	ISLA total CD4 T cells	161	12	11683803	R	ETV6	Opposite	NA			B1
Acute UD	PA14	ISLA total CD4 T cells	162	17	76765797	R	MFSD11	Opposite	NA			A1
Acute UD	PA14	ISLA total CD4 T cells	163	16	24750666	R	TNRC6A	Opposite	NA			A1
Acute UD	PA14	ISLA total CD4 T cells	164	1	23624251	R	Upstream: ID3 (64.457 kb); Downstream: MDS2 (3.083 kb)		Name: MIR; Class: SINE; Family: MIR			A1
Acute UD	PA14	ISLA total CD4 T cells	165	17	42332070	F	STAT3	Opposite	Name: AluSp; Class: SINE; Family: Alu			A1
Acute UD	PA14	ISLA total CD4 T cells	166	6	31628986	F	PRRC2A	Same	NA			A1
Acute UD	PA14	ISLA total CD4 T cells	167	22	41110024	F	EP300	Same	Name: AluSx1; Class: SINE; Family: Alu			A1
Acute UD	PA14	ISLA total CD4 T cells	168	17	2296681	F	SMG6	Opposite	Name: MER1B; Class: DNA; Family: hAT-Charlie			A1
Acute UD	PA14	ISLA total CD4 T cells	169	16	68279622	F	SLC7A6	Same	Name: AluSz; Class: SINE; Family: Alu			A1
Acute UD	PA14	ISLA total CD4 T cells	170	11	67623932	F	Upstream: DOC2GP (8.268 kb); Downstream: NUDT8 (4.006 kb)		Name: HERV9NC-int; Class: LTR; Family: ERV1			A1
Acute UD	PA14	ISLA total CD4 T cells	171	11	216982	F	SIRT3	Opposite	NA			A1
Acute UD	PA14	ISLA total CD4 T cells	172	19	5956550	F	RANBP3	Opposite	NA			A1
Acute UD	PA14	ISLA total CD4 T cells	173	4	169657618	R	CLCN3	Opposite	Name: MIR3; Class: SINE; Family: MIR			A2
Acute UD	PA14	ISLA total CD4 T cells	174	16	69305481	R	SNTB2	Opposite	NA			A1
Acute UD	PA14	ISLA total CD4 T cells	175	3	24211991	F	THRB	Opposite	Name: AluSq2; Class: SINE; Family: Alu			B3
Acute UD	PA14	ISLA total CD4 T cells	176	1	161102640	F	PFDN2	Opposite	Name: L2b; Class: LINE; Family: L2			A1
Acute UD	PA14	ISLA total CD4 T cells	177	5	177244509	R	NSD1	Opposite	NA			NA
Acute UD	PA14	ISLA total CD4 T cells	178	1	21246795	F	ECE1	Opposite	Name: AluJr; Class: SINE; Family: Alu			A1
Acute UD	PA14	ISLA total CD4 T cells	179	1	228472708	R	Upstream: HIST3H2BA (8.133 kb); Downstream: RNF187 (14.659 kb)		NA			B1
Acute UD	PA14	ISLA total CD4 T cells	180	8	27365648	R	PTK2B	Opposite	Name: MIR; Class: SINE; Family: MIR			A2
Acute UD	PA14	ISLA total CD4 T cells	181	12	123187832	F	MPHOSPH9	Opposite	NA			A1
Acute UD	PA14	ISLA total CD4 T cells	182	19	1121780	R	SBNO2	Same	NA			A1
Acute UD	PA14	ISLA total CD4 T cells	183	1	30971381	R	PUM1	Same	NA			B1
Acute UD	PA14	ISLA total CD4 T cells	184	2	239163342	F	HDAC4	Opposite	NA			A1
Acute UD	PA14	ISLA total CD4 T cells	185	13	20037589	R	ZMYM2	Opposite	NA			B2
Acute UD	PA14	ISLA total CD4 T cells	186	11	9418925	F	IPO7	Same	Name: L1ME2; Class: LINE; Family: L1			A1
Acute UD	PA14	ISLA total CD4 T cells	187	12	68615063	F	RAP1B	Same	NA			A2
Acute UD	PA14	ISLA total CD4 T cells	188	19	49579200	F	NOSIP	Opposite	Name: L1MC5a; Class: LINE; Family: L1			A1
Acute UD	PA14	ISLA total CD4 T cells	189	16	69032446	R	TANGO6	Opposite	Name: AluSx; Class: SINE; Family: Alu			A1
Acute UD	PA15	ISLA total CD4 T cells	190	19	38588875	R	MAP4K1	Same	Name: AluSx; Class: SINE; Family: Alu			B1
Acute UD	PA15	ISLA total CD4 T cells	191	3	52588397	F	PBRM1	Opposite	Name: L2b; Class: LINE; Family: L2			A1
Acute UD	PA15	ISLA total CD4 T cells	192	2	24512756	R	NCOA1	Opposite	NA			A1
Acute UD	PA15	ISLA total CD4 T cells	193	15	65017976	F	MTFMT	Opposite	Name: L1MC5a; Class: LINE; Family: L1			A1
Acute UD	PA15	ISLA total CD4 T cells	194	10	73456566	R	PPP3CB	Same	Name: L1MB8; Class: LINE; Family: L1			A1
Acute UD	PA15	ISLA total CD4 T cells	195	2	119774296	R	PTPN4	Opposite	Name: Tigger2; Class: DNA; Family: TcMar-Tigger			B2

Acute UD	PA15	ISLA total CD4 T cells	196	6	139171248	R	TXLNB	Mixed	NA			A2
Acute UD	PA15	ISLA total CD4 T cells	196	6	139171248	R	HECA		NA			
Acute UD	PA15	ISLA total CD4 T cells	197	5	80213846	R	SERINC5	Same	Name: L2b; Class: LINE; Family: L2			A2
Acute UD	PA15	ISLA total CD4 T cells	198	X	154129742	F	Upstream: MECP2 (32.011 kb); Downstream: OPN1LW (14.482 kb)		Name: AluSx1; Class: SINE; Family: Alu			
Acute UD	PA15	ISLA total CD4 T cells	199	6	30821928	R	LINC00243	Same	NA			A1
Acute UD	PA15	ISLA total CD4 T cells	200	4	3124596	R	HTT	Opposite	Name: Charlie15a; Class: DNA; Family: hAT-Charlie			A1
Acute UD	PA15	ISLA total CD4 T cells	201	4	2675915	R	FAM193A	Opposite	Name: L1MB3; Class: LINE; Family: L1			A1
Acute UD	PA15	ISLA total CD4 T cells	202	7	129690562	R	NRF1	Opposite	NA			A1
Acute UD	PA15	ISLA total CD4 T cells	203	8	134517354	R	ZFAT	Same	Name: L1MC5; Class: LINE; Family: L1			A2
Acute UD	PA15	ISLA total CD4 T cells	204	20	50515449	R	PTPN1	Opposite	NA			B1
Acute UD	PA15	ISLA total CD4 T cells	205	16	29004814	R	Upstream: LAT (14.031 kb); Downstream: LOC730153 (33.02 kb)		Name: HERVL-int; Class: LTR; Family: ERVL			A1
Acute UD	PA15	ISLA total CD4 T cells	206	11	128600190	R	LOC105369567	Same	NA			A2
Acute UD	PA15	ISLA total CD4 T cells	207	17	42606737	F	FAM134C	Opposite	Name: AluSq2; Class: SINE; Family: Alu			A1
Acute UD	PA15	ISLA total CD4 T cells	208	1	113951030	R	HIPK1	Opposite	Name: MER104; Class: DNA; Family: TcMar-Tc2			B1
Acute UD	PA15	ISLA total CD4 T cells	209	7	77747972	R	RSBN1L	Opposite	NA			B3
Acute UD	PA15	ISLA total CD4 T cells	210	5	179593831	R	RUFY1	Opposite	NA			B1
Acute UD	PA15	ISLA total CD4 T cells	211	2	112231513	R	ZC3H8	Same	NA			NA
Acute UD	PA15	ISLA total CD4 T cells	212	3	47131636	F	SETD2	Opposite	Name: AluY; Class: SINE; Family: Alu			A1
Acute UD	PA15	ISLA total CD4 T cells	213	1	226282354	R	LIN9	Same	Name: L1ME4b; Class: LINE; Family: L1			A2
Acute UD	PA15	ISLA total CD4 T cells	214	19	1086647	R	HMHA1	Mixed	NA			A1
Acute UD	PA15	ISLA total CD4 T cells	214	19	1086647	R	POLR2E		NA			
Acute UD	PA15	ISLA total CD4 T cells	215	9	15448291	R	SNAPC3	Opposite	NA			A2
Acute UD	PA15	ISLA total CD4 T cells	216	15	61959442	F	VPS13C	Opposite	NA			B1
Acute UD	PA15	ISLA total CD4 T cells	217	17	5474125	F	DERL2	Opposite	NA			A1
Acute UD	PA15	ISLA total CD4 T cells	218	5	151196686	F	CCDC69	Opposite	Name: L1MB5; Class: LINE; Family: L1			A2
Acute UD	PA15	ISLA total CD4 T cells	219	X	135308212	F	ZNF75D	Opposite	NA			NA
Acute UD	PA15	ISLA total CD4 T cells	220	17	8244136	F	CTC1	Opposite	NA			A1
Acute UD	PA15	ISLA total CD4 T cells	221	4	109821603	R	GAR1	Opposite	NA			B3
Acute UD	PA15	ISLA total CD4 T cells	222	9	131207014	R	NUP214	Opposite	Name: L2a; Class: LINE; Family: L2			A1
Acute UD	PA15	ISLA total CD4 T cells	223	12	11879773	F	ETV6	Same	NA			A2
Acute UD	PA15	ISLA total CD4 T cells	224	10	19396580	R	MALRD1	Opposite	Name: MIR; Class: SINE; Family: MIR			B2
Acute UD	PA15	ISLA total CD4 T cells	225	12	116089281	F	MED13L	Opposite	Name: FLAM_A; Class: SINE; Family: Alu			B1
Acute UD	PA19	ISLA total CD4 T cells	226	7	30606362	R	GARS	Opposite	Name: L1MC5; Class: LINE; Family: L1			A2
Acute UD	PA19	ISLA total CD4 T cells	227	1	66979667	R	MIER1	Opposite	NA			B3
Acute UD	PA19	ISLA total CD4 T cells	228	17	4287464	F	UBE2G1	Opposite	NA			A1
Acute UD	PA19	ISLA total CD4 T cells	229	12	123406646	F	SETD8	Same	NA			A1
Acute UD	PA19	ISLA total CD4 T cells	230	1	147957320	R	GPR89B	Opposite	Name: MIR; Class: SINE; Family: MIR			NA
Acute UD	PA19	ISLA total CD4 T cells	231	5	176262120	R	SIMC1	Opposite	Name: L1MC; Class: LINE; Family: L1			B1
Acute UD	PA19	ISLA total CD4 T cells	232	10	103168819	F	NT5C2	Opposite	NA			A1
Acute UD	PA19	ISLA total CD4 T cells	233	11	65439991	R	LOC105369345	Same	Name: FLAM_C; Class: SINE; Family: Alu			A1
Acute UD	PA19	ISLA total CD4 T cells	234	19	51745968	F	FPR1	Opposite	NA			B1
Acute UD	PA19	ISLA total CD4 T cells	235	5	179560941	R	RUFY1	Opposite	Name: AluSz; Class: SINE; Family: Alu			B1
Acute UD	PA19	ISLA total CD4 T cells	236	4	96696689	R	Upstream: LOC105377338 (65.451 kb); Downstream: LOC105377339 (153.698 kb)		Name: AluSp; Class: SINE; Family: Alu			B3
Acute UD	PA19	ISLA total CD4 T cells	237	17	77984094	R	Upstream: LOC105371909 (48.746 kb); Downstream: TNRC6C (20.076 kb)		Name: LTR41C; Class: LTR; Family: ERVL			A1
Acute UD	PA19	ISLA total CD4 T cells	238	11	128586892	R	ETS1	Same	NA			A2
Acute UD	PA19	ISLA total CD4 T cells	239	14	93256674	F	BTBD7	Opposite	Name: AluSx; Class: SINE; Family: Alu			A1
Acute UD	PA19	ISLA total CD4 T cells	240	7	150468624	R	GIMAP8	Opposite	NA			A2
Acute UD	PA19	ISLA total CD4 T cells	241	13	50518198	R	DLEU1	Opposite	Name: MIRb; Class: SINE; Family: MIR			A2
Acute UD	PA19	ISLA total CD4 T cells	242	8	143457352	F	ZC3H3	Opposite	Name: U5; Class: snRNA; Family: snRNA			A1
Acute UD	PA19	ISLA total CD4 T cells	243	11	73104349	F	FCHSD2	Opposite	Name: AluSz; Class: SINE; Family: Alu			A1
Acute UD	PA19	ISLA total CD4 T cells	244	3	69017810	F	Upstream: EOGT (3.849 kb); Downstream: TMF1 (2.017 kb)		Name: L1MC5; Class: LINE; Family: L1			A2
Acute UD	PA19	ISLA total CD4 T cells	245	20	3911282	R	PANK2	Opposite	NA			A1
Acute UD	PA19	ISLA total CD4 T cells	246	10	68982462	R	DDX21	Opposite	NA			B3
Acute UD	PA19	ISLA total CD4 T cells	247	3	111563398	R	CD96	Opposite	Name: L1M4a1; Class: LINE; Family: L1			B3
Acute UD	PA19	ISLA total CD4 T cells	248	16	31522466	R	Upstream: LOC101928829 (12.897 kb); Downstream: AHSP (5.386 kb)		Name: Charlie2a; Class: DNA; Family: hAT-Charlie			B1
Acute UD	PA19	ISLA total CD4 T cells	249	2	106142909	F	UXS1	Opposite	Name: (TG)n; Class: Simple_repeat; Family: Simple_repeat			B2
Acute UD	PA19	ISLA total CD4 T cells	250	2	42288719	F	EML4	Same	NA			B3
Acute UD	PA19	ISLA total CD4 T cells	251	16	31190776	R	FUS	Opposite	NA			A1
Acute UD	PA19	ISLA total CD4 T cells	252	16	29425827	F	SMG1P6	Opposite	NA			NA
Acute UD	PA19	ISLA total CD4 T cells	253	16	31895338	R	ZNF267	Opposite	Name: L1MA6; Class: LINE; Family: L1			B2
Acute UD	PA19	ISLA total CD4 T cells	254	4	6805829	F	KIAA0232	Same	NA			A1
Acute UD	PA19	ISLA total CD4 T cells	255	X	154523578	F	Upstream: FAM3A (7.331 kb); Downstream: G6PD (7.812 kb)		Name: AluSx1; Class: SINE; Family: Alu			NA
Acute UD	PA19	ISLA total CD4 T cells	256	22	29981508	R	MTMR3	Opposite	NA			A1
Acute UD	PA19	ISLA total CD4 T cells	257	15	41677691	R	MGA	Opposite	Name: L1M4; Class: LINE; Family: L1			A1
Acute UD	PA19	ISLA total CD4 T cells	258	13	40558216	F	FOXO1	Opposite	NA			A2
Acute UD	PA34	ISLA total CD4 T cells	259	12	49074483	F	Upstream: RHEBL1 (4.458 kb); Downstream: DHH (14.938 kb)		Name: AluJb; Class: SINE; Family: Alu			A1
Acute UD	PA34	ISLA total CD4 T cells	260	19	23269056	F	IPO5P1	Opposite	NA			B2
Acute UD	PA34	ISLA total CD4 T cells	261	14	103559272	R	BAG5	Same	NA			A1
Acute UD	PA34	ISLA total CD4 T cells	262	4	48733556	R	FRYL	Same	Name: L1MC1; Class: LINE; Family: L1			A2
Acute UD	PA34	ISLA total CD4 T cells	263	1	155469419	F	ASH1L	Opposite	Name: AluSp; Class: SINE; Family: Alu			A1
Acute UD	PA34	ISLA total CD4 T cells	264	14	22056539	R	TRA	Opposite	Name: L1PA16; Class: LINE; Family: L1			B1
Acute UD	PA34	ISLA total CD4 T cells	265	1	151115268	R	GABPB2	Opposite	Name: AluSp; Class: SINE; Family: Alu			A1
Acute UD	PA34	ISLA total CD4 T cells	266	9	77929889	F	GNAQ	Opposite	Name: L1ME4a; Class: LINE; Family: L1			B3
Acute UD	PA34	ISLA total CD4 T cells	267	9	112311665	F	PTBP3	Opposite	NA			B1
Acute UD	PA34	ISLA total CD4 T cells	268	17	77979582	F	Upstream: LOC105371909 (44.234 kb); Downstream: TNRC6C (24.588 kb)		Name: L1ME4c; Class: LINE; Family: L1			A1
Acute UD	PA34	ISLA total CD4 T cells	269	17	14114550	R	COX10	Opposite	NA			B2
Acute UD	PA34	ISLA total CD4 T cells	270	3	12654952	R	RAF1	Same	Name: FLAM_C; Class: SINE; Family: Alu			A1
Acute UD	PA34	ISLA total CD4 T cells	271	17	47911041	R	SP2	Mixed	Name: AluJb; Class: SINE; Family: Alu			A1
Acute UD	PA34	ISLA total CD4 T cells	271	17	47911041	R	SP2-AS1					
Acute UD	PA34	ISLA total CD4 T cells	272	19	50394439	F	POLD1	Same	Name: AluSx1; Class: SINE; Family: Alu			A1
Acute UD	PA34	ISLA total CD4 T cells	273	17	64118773	F	ERN1	Opposite	NA			B1
Acute UD	PA34	ISLA total CD4 T cells	274	10	67997075	F	HERC4	Opposite	NA			B3
Acute UD	PA34	ISLA total CD4 T cells	275	16	67090329	R	CBFB	Opposite	NA	x		A1
Acute UD	PA34	ISLA total CD4 T cells	276	16	67090329	R	CBFB	Opposite	NA	x		A1
Acute UD	PA34	ISLA total CD4 T cells	277	3	56734481	F	ARHGEF3	Opposite	Name: L1PA17; Class: LINE; Family: L1			A1
Acute UD	PA34	ISLA total CD4 T cells	278	17	61808436	F	BRIP1	Opposite	NA			A1
Acute UD	PA34	ISLA total CD4 T cells	279	6	158265220	R	TULP4	Opposite	Name: MIRc; Class: SINE; Family: MIR			A2
Acute UD	PA34	ISLA total CD4 T cells	280	14	71011693	F	PCNX	Same	NA			A1
Acute UD	PA34	ISLA total CD4 T cells	281	14	55117527	F	Upstream: MAPK1IP1L (47.333 kb); Downstream: LGALS3 (11.69 kb)		Name: AluSz; Class: SINE; Family: Alu			A2
Acute UD	PA34	ISLA total CD4 T cells	282	Y	13448383	F	UTY	Opposite	Name: L1M4; Class: LINE; Family: L1			NA
Acute UD	PA34	ISLA total CD4 T cells	283	4	77765418	R	CNOT6L	Same	Name: L1ME3E; Class: LINE; Family: L1			A2
Acute UD	PA34	ISLA total CD4 T cells	284	17	35627632	R	AP2B1	Opposite	NA			A1
Acute UD	PA34	ISLA total CD4 T cells	285	20	3335856	R	C20orf194	Same	Name: AluSz6; Class: SINE; Family: Alu			A1
Acute UD	PA34	ISLA total CD4 T cells	286	21	42241416	R	ABCG1	Opposite	Name: AluSz; Class: SINE; Family: Alu			B2
Acute UD	PA34	ISLA total CD4 T cells	287	16	11793398	F	ZC3H7A	Opposite	Name: AluJr4; Class: SINE; Family: Alu			A1
Acute UD	PA34	ISLA total CD4 T cells	288	16	89475707	F	ANKRD11	Opposite	NA			A1
Acute UD	PA34	ISLA total CD4 T cells	289	3	129294688	R	HMCES	Opposite	Name: L1MEc; Class: LINE; Family: L1			A1
Acute UD	PA34	ISLA total CD4 T cells	290	3	185182739	F	EHHADH-AS1	Same	Name: ERV3-16A3_I-int; Class: LTR; Family: ERVL			A2
Acute UD	PA34	ISLA total CD4 T cells	291	10	123005013	F	IKZF5	Opposite	Name: FLAM_C; Class: SINE; Family: Alu			B1
Acute UD	PA34	ISLA total CD4 T cells	292	13	42312666	R	AKAP11	Opposite	NA			A2
Acute UD	PA34	ISLA total CD4 T cells	293	7	101876984	R	CUX1	Opposite	Name: L1M5; Class: LINE; Family: L1			A1
Acute UD	PA34	ISLA total CD4 T cells	294	17	16213302	F	NCOR1	Opposite	Name: AluSc8; Class: SINE; Family: Alu			A1
Acute UD	PA34	ISLA total CD4 T cells	295	17	39779821	F	IKZF3	Opposite	NA			A1
Acute UD	PA34	ISLA total CD4 T cells	296	15	66429482	R	MAP2K1	Opposite	Name: L1ME3A; Class: LINE; Family: L1			A1
Acute UD	PA34	ISLA total CD4 T cells	297	2	171141188	R	TLK1	Same	Name: L1MEg; Class: LINE; Family: L1			A2
Acute UD	PA34	ISLA total CD4 T cells	298	1	150741419	F	CTSS	Opposite	Name: L1ME4a; Class: LINE; Family: L1			A1
Acute UD	PA34	ISLA total CD4 T cells	299	21	10463805	R	BAGE2	Opposite	NA			NA
Acute UD	PA34	ISLA total CD4 T cells	299	21	10463805	R	LOC105378260	Opposite	NA			NA
Acute UD	PA34	ISLA total CD4 T cells	300	X	123614716	R	THOC2	Same	NA			NA
Acute UD	PA34	ISLA total CD4 T cells	301	22	41114115	R	EP300	Opposite	NA			A1
Acute UD	PA34	ISLA total CD4 T cells	302	2	44553903	F	CAMKMT	Same	NA			A2
Acute UD	PA34	ISLA total CD4 T cells	303	10	133408497	R	MTG1	Opposite	Name: L1MA4; Class: LINE; Family: L1			B2
Acute UD	PA34	ISLA total CD4 T cells	304	19	13971205	R	RFX1	Same	Name: AluSq2; Class: SINE; Family: Alu			A1
Acute UD	PA34	ISLA total CD4 T cells	305	16	80640336	F	CDYL2	Opposite	Name: L1MA2; Class: LINE; Family: L1			B1
Acute UD	PA34	ISLA total CD4 T cells	306	17	82931922	R	TBCD	Opposite	NA			NA
Acute UD	PA34	ISLA total CD4 T cells	307	19	1272771	R	CIRBP	Opposite	NA			A1
Acute UD	PA34	ISLA total CD4 T cells	308	6	32836127	F	TAP2	Opposite	NA			A1
Acute UD	PA34	ISLA total CD4 T cells	309	16	50324274	F	BRD7	Opposite	Name: L2a; Class: LINE; Family: L2			A2
Acute UD	PA34	ISLA total CD4 T cells	310	8	73859736	F	UBE2W	Opposite	Name: Tigger2a; Class: DNA; Family: TcMar-Tigger			B3
Acute UD	PA34	ISLA total CD4 T cells	311	6	42062783	F	TAF8	Same	Name: AluSg4; Class: SINE; Family: Alu			A1
Acute UD	PA34	ISLA total CD4 T cells	312	9	3479658	F	RFX3	Opposite	NA			A2
Acute UD	PA34	ISLA total CD4 T cells	313	17	80717194	R	RPTOR	Opposite	Name: L2a; Class: LINE; Family: L2			A1
Acute UD	PA34	ISLA total CD4 T cells	314	8	47903873	R	PRKDC	Same	NA			B2
Acute UD	PA34	ISLA total CD4 T cells	315	1	154266021	F	UBAP2L	Same	NA			A1
Acute UD	PA34	ISLA total CD4 T cells	316	11	62851485	F	Upstream: RNU2-2P (9.724 kb); Downstream: SNHG1 (0.503 kb)		Name: AluSx1; Class: SINE; Family: Alu			B1
Acute UD	PA34	ISLA total CD4 T cells	317	16	79265486	R	MAF	Same	Name: MER44C; Class: DNA; Family: TcMar-Tigger			B2
Acute UD	PA34	ISLA total CD4 T cells	318	3	88026934	F	Upstream: HTR1F (32.078 kb); Downstream: CGGBP1 (25.016 kb)		Name: LTR16C; Class: LTR; Family: ERVL			B3
Acute UD	PA34	ISLA total CD4 T cells	319	6	30231790	F	Upstream: TRIM26 (18.296 kb); Downstream: HCG17 (2.249 kb)		Name: MLT1J1-int; Class: LTR; Family: ERVL-MaLR			A1
Acute UD	PA34	ISLA total CD4 T cells	320	6	17680931	F	NUP153	Opposite	Name: L1MA4; Class: LINE; Family: L1			A2
Acute UD	PA34	ISLA total CD4 T cells	321	16	57221446	F	RSPRY1	Same	NA			A1
Acute UD	PA34	ISLA total CD4 T cells	322	17	2080516	F	SMG6	Opposite	NA			A1
Acute UD	PA34	ISLA total CD4 T cells	323	8	22473864	R	PPP3CC	Opposite	NA			A1
Acute UD	PA34	ISLA total CD4 T cells	324	12	131504412	R	LOC101929974	Same	Name: L1PA5; Class: LINE; Family: L1			A2
Acute UD	PA34	ISLA total CD4 T cells	325	20	37812817	R	CTNNBL1	Opposite	Name: L2c; Class: LINE; Family: L2			B1
Acute UD	PA34	ISLA total CD4 T cells	326	16	57007188	F	NLRC5	Same	Name: L1ME4b; Class: LINE; Family: L1			A1
Acute UD	PA34	ISLA total CD4 T cells	327	16	17385915	R	XYLT1	Same	Name: MIR; Class: SINE; Family: MIR			A1
Acute UD	PA34	ISLA total CD4 T cells	328	17	5323933	F	RABEP1	Same	Name: L1MA4; Class: LINE; Family: L1			A1
Acute UD	PA34	ISLA total CD4 T cells	329	9	131689534	R	RAPGEF1	Same	Name: MER65-int; Class: LTR; Family: ERV1			A1
Acute UD	PA34	ISLA total CD4 T cells	330	21	44784880	F	UBE2G2	Opposite	NA			A1
Acute UD	PA34	ISLA total CD4 T cells	331	19	22065562	R	ZNF257	Opposite	NA		x	B2
Acute UD	PA35	ISLA total CD4 T cells	332	2	197564396	R	Upstream: MOB4 (10.697 kb); Downstream: RFTN2 (6.404 kb)		Name: L1PA6; Class: LINE; Family: L1			A2
Acute UD	PA35	ISLA total CD4 T cells	333	5	176478083	R	FAF2	Opposite	NA			A1
Acute UD	PA35	ISLA total CD4 T cells	334	X	41865029	R	CASK	Same	NA			NA
Acute UD	PA35	ISLA total CD4 T cells	335	16	2296325	F	ABCA3	Opposite	Name: AluSz; Class: SINE; Family: Alu			A1
Acute UD	PA35	ISLA total CD4 T cells	336	5	177183439	R	NSD1	Opposite	Name: L1M4; Class: LINE; Family: L1			NA

Acute UD	PA35	ISLA total CD4 T cells	337	15	42201053	F	VPS39	Opposite	Name: L1MB8; Class: LINE; Family: L1			A1
Acute UD	PA35	ISLA total CD4 T cells	338	19	50035179	R	ZNF473	Opposite	Name: AluJb; Class: SINE; Family: Alu		x	A1
Acute UD	PA35	ISLA total CD4 T cells	339	17	78060945	R	TNRC6C	Opposite	NA			A1
Acute UD	PA35	ISLA total CD4 T cells	340	2	9534731	F	ADAM17	Opposite	Name: L1ME4a; Class: LINE; Family: L1			A2
Acute UD	PA35	ISLA total CD4 T cells	341	17	76186520	F	RNF157	Opposite	Name: AluSq; Class: SINE; Family: Alu			A1
Acute UD	PA35	ISLA total CD4 T cells	342	17	35259718	R	SLFN5	Opposite	NA			A1
Acute UD	PA35	ISLA total CD4 T cells	343	10	133407075	R	MTG1	Opposite	Name: L1MA4; Class: LINE; Family: L1			B2
Acute UD	PA35	ISLA total CD4 T cells	344	12	56182784	F	SMARCC2	Opposite	me: (CATAAT)n; Class: Simple_repeat; Family: Simple_repeat			A1
Acute UD	PA35	ISLA total CD4 T cells	345	Y	11200090	R	LOC105379273	Same	Name: MLT1H1; Class: LTR; Family: ERVL-MaLR			NA
Acute UD	PA35	ISLA total CD4 T cells	346	2	167428159	F	B3GALT1	Same	NA			B3
Acute UD	PA35	ISLA total CD4 T cells	347	3	16983074	F	PLCL2	Same	NA			A2
Acute UD	PA35	ISLA total CD4 T cells	348	12	128587381	F	TMEM132C	Same	Name: MLT1A0; Class: LTR; Family: ERVL-MaLR			B2
Acute UD	PA35	ISLA total CD4 T cells	349	17	1386165	F	YWHAE	Opposite	NA			A1
Acute UD	PA35	ISLA total CD4 T cells	350	11	67163107	R	KDM2A	Opposite	NA			A1
Acute UD	PA35	ISLA total CD4 T cells	351	14	21725914	R	TRA	Opposite	Name: L1MA8; Class: LINE; Family: L1			A1
Acute UD	PA35	ISLA total CD4 T cells	352	11	118835789	F	Upstream: SETP16 (1.224 kb); Downstream: CXCR5 (47.977 kb)		Name: AluY; Class: SINE; Family: Alu			A1
Acute UD	PA35	ISLA total CD4 T cells	353	3	17379118	R	TBC1D5	Same	NA			A2
Acute UD	PA35	ISLA total CD4 T cells	354	20	31680877	F	LOC105372589	Mixed	Name: L2a; Class: LINE; Family: L2			B1
Acute UD	PA35	ISLA total CD4 T cells	354	20	31680877	F	BCL2L1					
Acute UD	PA35	ISLA total CD4 T cells	355	16	30489390	R	ITGAL	Opposite	NA			A1
Acute UD	PA35	ISLA total CD4 T cells	356	11	110250561	R	RDX	Same	Name: OldhAT1; Class: DNA; Family: hAT-Ac			A2
Acute UD	PA35	ISLA total CD4 T cells	357	12	93496334	R	MRPL42	Opposite	Name: AluSz; Class: SINE; Family: Alu			A2
Acute UD	PA35	ISLA total CD4 T cells	358	12	67286231	R	CAND1	Opposite	Name: AluYm1; Class: SINE; Family: Alu			B3
Acute UD	PA35	ISLA total CD4 T cells	359	1	25481792	R	TMEM57	Opposite	NA			A1
Acute UD	PA35	ISLA total CD4 T cells	360	3	42604140	R	NKTR	Opposite	Name: L1MEh; Class: LINE; Family: L1			A1
Acute UD	PA35	ISLA total CD4 T cells	361	20	35711215	F	RBM39	Opposite	NA			A1
Acute UD	PA35	ISLA total CD4 T cells	362	8	44127282	R	Upstream: SNX18P27 (612.861 kb); Downstream: ASNSP1 (2451.924 kb)		Name: ALR/Alpha; Class: Satellite; Family: centr			NA
Acute UD	PA35	ISLA total CD4 T cells	363	16	50760997	F	CYLD	Same	Name: L1ME4b; Class: LINE; Family: L1			A2
Acute UD	PA35	ISLA total CD4 T cells	364	6	142190671	R	VTA1	Opposite	Name: L1M5; Class: LINE; Family: L1			B3
Acute UD	PA35	ISLA total CD4 T cells	365	7	37166130	R	ELMO1	Same	Name: L1PA3; Class: LINE; Family: L1			A2
Acute UD	PA35	ISLA total CD4 T cells	366	16	30302272	F	SMG1P5	Opposite	NA			A1
Acute UD	PA35	ISLA total CD4 T cells	367	19	14109343	F	PRKACA	Opposite	Name: AluSx1; Class: SINE; Family: Alu			A1
Acute UD	PA35	ISLA total CD4 T cells	368	2	48319474	R	FOXN2	Opposite	NA			A2
Acute UD	PA35	ISLA total CD4 T cells	369	17	31167942	R	NF1	Opposite	Name: L2a; Class: LINE; Family: L2			A1
Acute UD	PA35	ISLA total CD4 T cells	370	8	26305957	R	PPP2R2A	Opposite	NA			B1
Acute UD	PA35	ISLA total CD4 T cells	371	17	42473109	F	ATP6V0A1	Same	NA			A1
Acute UD	PA35	ISLA total CD4 T cells	372	11	118462052	R	KMT2A	Opposite	Name: AluSx; Class: SINE; Family: Alu			A1
Acute UD	PA35	ISLA total CD4 T cells	373	22	40835855	R	ST13	Same	NA			A1
Acute UD	PA35	ISLA total CD4 T cells	374	10	33072310	F	LOC101929475	Same	Name: MER4D1; Class: LTR; Family: ERV1			B2
Acute UD	PA35	ISLA total CD4 T cells	375	5	44823152	R	Upstream: MRPS30 (7.638 kb); Downstream: LOC105374751 (403.372 kb)		NA			B2
Acute UD	PA35	ISLA total CD4 T cells	376	1	204506599	R	TRK-TTT3-1	Opposite	Name: tRNA-Lys-AAA; Class: tRNA; Family: tRNA			A1
Acute UD	PA35	ISLA total CD4 T cells	377	16	31308207	R	ITGAM	Opposite	Name: L1PA4; Class: LINE; Family: L1			B1
Acute UD	PA35	ISLA total CD4 T cells	378	6	16134026	R	MYLIP	Opposite	Name: L2a; Class: LINE; Family: L2			A2
Acute UD	PA35	ISLA total CD4 T cells	379	13	108235843	R	Upstream: ABHD13 (1.588 kb); Downstream: LOC105370354 (7.147 kb)		NA			B3
Acute UD	PA35	ISLA total CD4 T cells	380	5	62331242	R	KIF2A	Opposite	Name: AluSp; Class: SINE; Family: Alu			B3
Acute UD	PA35	ISLA total CD4 T cells	381	11	62388495	R	ASRGL1	Opposite	Name: AluSx; Class: SINE; Family: Alu			A1
Acute UD	PA35	ISLA total CD4 T cells	382	X	149623224	F	TMEM185A	Opposite	NA			NA
Acute UD	PA35	ISLA total CD4 T cells	383	19	10179668	F	DNMT1	Opposite	NA			B1
Acute UD	PA35	ISLA total CD4 T cells	384	12	877648	F	WNK1	Same	NA			A1
Acute UD	PA35	ISLA total CD4 T cells	385	12	62507217	R	MON2	Opposite	NA			B2
Acute UD	PA35	ISLA total CD4 T cells	386	22	40071053	F	TNRC6B	Same	Name: MER46C; Class: DNA; Family: TcMar-Tigger			A1
Acute UD	PA35	ISLA total CD4 T cells	387	16	29571491	F	Upstream: SMG1P2 (5.272 kb); Downstream: LOC105371168 (26.3 kb)		Name: MER77B; Class: LTR; Family: ERVL			NA
Acute UD	PA35	ISLA total CD4 T cells	388	17	20181529	R	SPECC1	Opposite	NA			B1
Acute UD	PA35	ISLA total CD4 T cells	389	5	59117933	F	PDE4D	Opposite	NA			B3
Acute UD	PA35	ISLA total CD4 T cells	390	5	36155972	R	SKP2	Opposite	NA			A2
Acute UD	PA35	ISLA total CD4 T cells	391	19	16363738	F	EPS15L1	Opposite	NA			A1
Acute UD	PA35	ISLA total CD4 T cells	392	1	149204855	F	Upstream: LOC653513 (3.709 kb); Downstream: LOC100996761 (40.949 kb)		Name: MIR3; Class: SINE; Family: MIR			B1
Acute UD	PA35	ISLA total CD4 T cells	393	5	148880835	R	Upstream: ADRB2 (52.201 kb); Downstream: SH3TC2 (101.315 kb)		Name: L2b; Class: LINE; Family: L2			A2
Acute UD	PA35	ISLA total CD4 T cells	394	19	49669904	R	BCL2L12	Opposite	NA			A1
Acute UD	PA35	ISLA total CD4 T cells	395	7	150521152	F	Upstream: GIMAP7 (0.079 kb); Downstream: ALDH7A1P3 (20.48 kb)		Name: L1MA9; Class: LINE; Family: L1			B1
Acute UD	PA35	ISLA total CD4 T cells	396	10	103004092	R	CNNM2	Opposite	Name: AluSx1; Class: SINE; Family: Alu			A1
Acute UD	PA35	ISLA total CD4 T cells	397	17	7488001	R	POLR2A	Opposite	Name: L2a; Class: LINE; Family: L2			A1
Acute UD	PA35	ISLA total CD4 T cells	398	5	72838698	R	TNPO1	Opposite	NA			A2
Acute UD	PA35	ISLA total CD4 T cells	399	5	148880474	R	Upstream: ADRB2 (51.84 kb); Downstream: SH3TC2 (101.676 kb)		Name: L2b; Class: LINE; Family: L2			A2
Acute UD	PA35	ISLA total CD4 T cells	400	11	67407002	F	TBC1D10C	Same	NA			A1
Acute UD	PA35	ISLA total CD4 T cells	401	15	18068743	R	Upstream: N/A (0 kb); Downstream: LOC101059971 (1829.452 kb)		Name: ALR/Alpha; Class: Satellite; Family: centr			NA
Acute UD	PA35	ISLA total CD4 T cells	402	11	118325352	F	Upstream: CD3E (9.177 kb); Downstream: CD3D (13.722 kb)		Name: L1PB1; Class: LINE; Family: L1			A1
Acute UD	PA35	ISLA total CD4 T cells	403	11	67133128	R	KDM2A	Opposite	Name: AluSq2; Class: SINE; Family: Alu			A1
Acute UD	PA35	ISLA total CD4 T cells	404	8	130284532	F	ASAP1	Opposite	NA			B3
Acute UD	PA35	ISLA total CD4 T cells	405	17	39509552	R	CDK12	Opposite	NA			B1
Acute UD	PA35	ISLA total CD4 T cells	406	Unknown	100229	R	Upstream: N/A (0 kb); Downstream: LOC101930589 (123.83 kb)		NA			NA
Acute UD	PA35	ISLA total CD4 T cells	407	6	33308763	F	TAPBP	Opposite	Name: MER30; Class: DNA; Family: hAT-Charlie			A1
Acute UD	PA35	ISLA total CD4 T cells	408	13	79527616	R	NDFIP2	Opposite	Name: Tigger3b; Class: DNA; Family: TcMar-Tigger			B3
Acute UD	PA35	ISLA total CD4 T cells	409	Unknown	100265	R	Upstream: N/A (0 kb); Downstream: LOC101930589 (123.794 kb)		NA			NA
Acute UD	PA35	ISLA total CD4 T cells	410	16	30285320	R	SMG1P5	Same	NA			NA
Acute UD	PA35	ISLA total CD4 T cells	411	19	41544993	F	Upstream: LINC01480 (8.089 kb); Downstream: CEACAM21 (4.257 kb)		Name: LTR5A; Class: LTR; Family: ERVK			B1
Acute UD	PA35	ISLA total CD4 T cells	412	1	147040762	F	LOC728989	Opposite	NA			B1
Acute UD	PA35	ISLA total CD4 T cells	413	8	76976182	F	Upstream: MIR3149 (9.332 kb); Downstream: PEX2 (4.076 kb)		Name: L1PA16; Class: LINE; Family: L1			B3
Acute UD	PA35	ISLA total CD4 T cells	414	13	46181069	F	LCP1	Opposite	NA			B1
Acute UD	PA35	ISLA total CD4 T cells	415	14	100070986	F	EVL	Same	NA			B1
Acute UD	PA35	ISLA total CD4 T cells	416	12	49398681	F	SPATS2	Same	NA			A1
Acute UD	PA35	ISLA total CD4 T cells	417	11	67273459	R	ADRBK1	Mixed	NA			A1
Acute UD	PA35	ISLA total CD4 T cells	417	11	67273459	R	LOC105369356		NA			
Acute UD	PA35	ISLA total CD4 T cells	418	7	134798461	F	CALD1	Mixed	NA			A2
Acute UD	PA35	ISLA total CD4 T cells	418	7	134798461	F	LOC102724947		NA			
Acute UD	PA35	ISLA total CD4 T cells	419	19	46967280	F	ARHGAP35	Same	NA			B1
Acute UD	PA35	ISLA total CD4 T cells	420	16	29078405	F	RRN3P2	Same	NA			A1
Acute UD	PA35	ISLA total CD4 T cells	421	17	78045743	R	TNRC6C	Opposite	NA			A1
Acute UD	PA35	ISLA total CD4 T cells	422	16	3555466	R	NLRC3	Mixed	Name: L1MB5; Class: LINE; Family: L1			A1
Acute UD	PA35	ISLA total CD4 T cells	422	16	3555466	R	LOC101929732					
Acute UD	PA35	ISLA total CD4 T cells	423	1	33331040	F	PHC2	Opposite	NA			A1
Acute UD	PA35	ISLA total CD4 T cells	424	6	36600064	R	SRSF3	Opposite	NA			A1
Acute UD	PA35	ISLA total CD4 T cells	425	19	2533925	F	GNG7	Opposite	NA			A1
Acute UD	PA35	ISLA total CD4 T cells	426	13	26306531	F	CDK8	Same	NA			B2
Acute UD	PA35	ISLA total CD4 T cells	427	21	8042993	F	Upstream: LOC105379505 (186.108 kb); Downstream: MIR6724-1 (162.322 kb)		Name: ALR/Alpha; Class: Satellite; Family: centr			NA
Acute UD	PA35	ISLA total CD4 T cells	428	9	136031281	F	NACC2	Opposite	Name: L1ME1; Class: LINE; Family: L1			A1
Acute UD	PA35	ISLA total CD4 T cells	429	16	47119113	F	NETO2	Opposite	Name: L1MEd; Class: LINE; Family: L1			A2
Acute UD	PA35	ISLA total CD4 T cells	430	17	4865231	R	MINK1	Opposite	Name: AluSx1; Class: SINE; Family: Alu			A1
Acute UD	PA35	ISLA total CD4 T cells	431	19	6812842	R	VAV1	Opposite	lame: (GAT)n; Class: Simple_repeat; Family: Simple_repeat			A1
Acute UD	PA35	ISLA total CD4 T cells	432	11	66118486	R	PACS1	Opposite	Name: AluJo; Class: SINE; Family: Alu			A1
Acute UD	PA35	ISLA total CD4 T cells	433	17	49291044	F	ZNF652	Opposite	NA			A1
Acute UD	PA38	ISLA total CD4 T cells	434	9	125759928	R	PBX3	Opposite	NA			A1
Acute UD	PA38	ISLA total CD4 T cells	435	19	57211916	R	ZNF264	Opposite	NA		x	B2
Acute UD	PA38	ISLA total CD4 T cells	436	17	37204863	R	ACACA	Same	NA			A1
Acute UD	PA38	ISLA total CD4 T cells	437	7	44624623	F	OGDH	Same	NA			A1
Acute UD	PA38	ISLA total CD4 T cells	438	1	9673452	R	PIK3CD	Mixed	Name: AluSz; Class: SINE; Family: Alu			A1
Acute UD	PA38	ISLA total CD4 T cells	438	1	9673452	R	PIK3CD-AS2					
Acute UD	PA38	ISLA total CD4 T cells	439	19	3972883	F	Upstream: DAPK3 (1.76 kb); Downstream: EEF2 (3.173 kb)		Name: AluSz; Class: SINE; Family: Alu			A1
Acute UD	PA38	ISLA total CD4 T cells	440	17	40150794	R	CASC3	Opposite	Name: AluY; Class: SINE; Family: Alu			A1
Acute UD	PA38	ISLA total CD4 T cells	441	2	68784659	F	ARHGAP25	Same	NA			A2
Acute UD	PA38	ISLA total CD4 T cells	442	9	20491168	F	MLLT3	Opposite	NA			A2
Acute UD	PA38	ISLA total CD4 T cells	443	10	101469843	R	BTRC	Opposite	Name: L1ME2; Class: LINE; Family: L1			A1
Acute UD	PA38	ISLA total CD4 T cells	444	3	49853168	F	TRAIP	Opposite	Name: L1MEd; Class: LINE; Family: L1			A1
Acute UD	PA38	ISLA total CD4 T cells	445	21	46340925	R	PCNT	Opposite	Name: AluSz; Class: SINE; Family: Alu			A1
Acute UD	PA38	ISLA total CD4 T cells	446	20	45018801	F	STK4	Same	Name: AluJr; Class: SINE; Family: Alu			A1
Acute UD	PA38	ISLA total CD4 T cells	447	6	35901526	F	SRPK1	Opposite	Name: MLT1B; Class: LTR; Family: ERVL-MaLR			A1
Acute UD	PA38	ISLA total CD4 T cells	448	6	73768521	R	CD109	Opposite	NA			B3
Acute UD	PA38	ISLA total CD4 T cells	449	14	76191845	F	GPATCH2L	Same	Name: L1MA5A; Class: LINE; Family: L1			A1
Acute UD	PA38	ISLA total CD4 T cells	450	22	40510290	F	MKL1	Opposite	NA			A1
Acute UD	PA38	ISLA total CD4 T cells	451	2	32507305	F	BIRC6	Same	Name: AluSz6; Class: SINE; Family: Alu			A2
Acute UD	PA38	ISLA total CD4 T cells	452	17	78685744	R	CYTH1	Same	Name: HAL1b; Class: LINE; Family: L1			A1
Acute UD	PA02	ISLA on p24+ cells	1	1	160580536	R	Upstream: CD84 (1.02 kb); Downstream: SLAMF1 (27.575 kb)		NA			
Acute UD	PA02	ISLA on p24+ cells	2	15	60797139	F	RORA	Opposite	Name: L1MB5; Class: LINE; Family: L1			
Acute UD	PA02	ISLA on p24+ cells	3	9	35631255	R	CD72	Same	Name: L1MEj; Class: LINE; Family: L1			
Acute UD	PA02	ISLA on p24+ cells	4	16	50286330	F	ADCY7	Same	Name: AluJb; Class: SINE; Family: Alu			
Acute UD	PA02	ISLA on p24+ cells	5	6	31553386	F	NFKBIL1	Mixed	Name: L1M5; Class: LINE; Family: L1	x		
Acute UD	PA02	ISLA on p24+ cells	5	6	31553386	F	LOC100287329					
Acute UD	PA02	ISLA on p24+ cells	6	6	31553386	F	NFKBIL1	Mixed	Name: L1M5; Class: LINE; Family: L1	x		
Acute UD	PA02	ISLA on p24+ cells	6	6	31553386	F	LOC100287329					
Acute UD	PA02	ISLA on p24+ cells	7	3	50597962	F	Upstream: HEMK1 (8.912 kb); Downstream: LOC105377084 (1.021 kb)		Name: AluJr; Class: SINE; Family: Alu			
Acute UD	PA02	ISLA on p24+ cells	8	17	48336471	F	SKAP1	Opposite	Name: L3b; Class: LINE; Family: CR1			
Acute UD	PA02	ISLA on p24+ cells	9	19	18957503	F	Upstream: LOC102724360 (10.667 kb); Downstream: SUGP2 (33.385 kb)		NA			
Acute UD	PA02	ISLA on p24+ cells	10	20	38855731	F	PPP1R16B	Same	NA			
Acute UD	PA02	ISLA on p24+ cells	11	19	3542749	R	C19orf71	Mixed	Name: AluSx1; Class: SINE; Family: Alu			
Acute UD	PA02	ISLA on p24+ cells	11	19	3542749	R	MFSD12					
Acute UD	PA14	ISLA on p24+ cells	12	Y	2972156	F	ZFY	Same	Name: Charlie21a; Class: DNA; Family: hAT-Charlie			
Acute UD	PA14	ISLA on p24+ cells	13	17	1839424	R	RPA1	Opposite	Name: AluSx1; Class: SINE; Family: Alu			
Acute UD	PA14	ISLA on p24+ cells	14	19	13169436	F	Upstream: RPL12P42 (10.562 kb); Downstream: CACNA1A (37.006 kb)		Name: AluSz; Class: SINE; Family: Alu			
Acute UD	PA14	ISLA on p24+ cells	15	17	75790330	R	UNK	Opposite	NA			
Acute UD	PA14	ISLA on p24+ cells	16	1	2240997	R	SKI	Opposite	NA			
Acute UD	PA14	ISLA on p24+ cells	17	2	64129094	F	PELI1	Opposite	NA			
Acute UD	PA14	ISLA on p24+ cells	18	7	100206687	F	GATS	Mixed	Name: AluSg; Class: SINE; Family: Alu			
Acute UD	PA14	ISLA on p24+ cells	18	7	100206687	F	STAG3					
Acute UD	PA14	ISLA on p24+ cells	19	19	41338574	F	TGFB1	Opposite	Name: AluSx1; Class: SINE; Family: Alu			
Acute UD	PA14	ISLA on p24+ cells	20	12	57283918	F	R3HDM2	Opposite	NA			

Acute UD	PA14	ISLA on p24+ cells	21	1	55119014	R	USP24	Same	Name: L1MB3; Class: LINE; Family: L1			
Acute UD	PA14	ISLA on p24+ cells	22	20	37052966	R	RBL1	Same	Name: L1MDa; Class: LINE; Family: L1			
Acute UD	PA14	ISLA on p24+ cells	23	1	120882007	R	LOC105378945	Opposite	Name: L1MEd; Class: LINE; Family: L1			
Acute UD	PA14	ISLA on p24+ cells	24	13	94574459	F	TGDS	Opposite	NA			
Acute UD	PA14	ISLA on p24+ cells	25	11	9198712	F	DENND5A	Opposite	Name: Tigger13a; Class: DNA; Family: TcMar-Tigger			
Acute UD	PA14	ISLA on p24+ cells	26	20	36988193	R	Upstream: SAMHD1 (36.35 kb); Downstream: RBL1 (8.156 kb)		NA			
Acute UD	PA14	ISLA on p24+ cells	27	13	114244793	F	CDC16	Same	NA			
Acute UD	PA14	ISLA on p24+ cells	28	2	191197284	F	STAT4	Mixed	NA			
Acute UD	PA14	ISLA on p24+ cells	28	2	191197284	F	LOC105373804		NA			
Acute UD	PA14	ISLA on p24+ cells	29	11	64771668	F	SF1	Opposite	NA			
Acute UD	PA14	ISLA on p24+ cells	30	1	198216279	R	NEK7	Opposite	Name: L1PB3; Class: LINE; Family: L1			
Acute UD	PA14	ISLA on p24+ cells	31	6	31786052	R	VARS	Same	Name: AluSx; Class: SINE; Family: Alu			
Acute UD	PA14	ISLA on p24+ cells	32	5	16610819	R	FAM134B	Same	Name: L1PA5; Class: LINE; Family: L1			
Acute UD	PA14	ISLA on p24+ cells	33	17	64106389	F	ERN1	Opposite	NA			
Acute UD	PA14	ISLA on p24+ cells	34	1	155006795	R	ZBTB7B	Opposite	NA			
Acute UD	PA34	ISLA on p24+ cells	35	17	77158520	R	SEC14L1	Opposite	NA			
Acute UD	PA34	ISLA on p24+ cells	36	1	39002500	R	AKIRIN1	Opposite	Name: AluY; Class: SINE; Family: Alu			
Acute UD	PA34	ISLA on p24+ cells	37	17	40382129	F	Upstream: LOC100421674 (0.291 kb); Downstream: TOP2A (6.392 kb)		Name: AluSz; Class: SINE; Family: Alu			
Acute UD	PA34	ISLA on p24+ cells	38	21	8241204	R	LOC102723570	Same	NA			
Acute UD	PA34	ISLA on p24+ cells	38	21	8424228	R	LOC102723584	Same	NA			
Acute UD	PA34	ISLA on p24+ cells	38	21	8468788	R	LOC102723603	Same	NA			
Acute UD	PA34	ISLA on p24+ cells	39	22	22992297	R	LOC105372948	Same	NA			
Acute UD	PA34	ISLA on p24+ cells	40	21	8228149	F	Upstream: LOC105379506 (4.687 kb); Downstream: LOC102723570 (12.59 kb)		Name: (TCTC)n; Class: Simple_repeat; Family: Simple_repeat			
Acute UD	PA34	ISLA on p24+ cells	40	21	8411148	F	Upstream: LOC105379508 (4.687 kb); Downstream: LOC102723584 (12.636 kb)		Name: (TCTC)n; Class: Simple_repeat; Family: Simple_repeat			
Acute UD	PA34	ISLA on p24+ cells	40	21	8455295	F	Upstream: LOC105379507 (4.647 kb); Downstream: LOC102723603 (11.535 kb)		Name: (TCTC)n; Class: Simple_repeat; Family: Simple_repeat			
Acute UD	PA34	ISLA on p24+ cells	41	18	8091246	R	PTPRM	Opposite	NA			
Acute UD	PA34	ISLA on p24+ cells	42	9	92853989	R	ZNF484	Same	Name: LTR52; Class: LTR; Family: ERVL			
Acute UD	PA34	ISLA on p24+ cells	43	7	59549476	F	Upstream: LOC102724116 (1460.891 kb); Downstream: LOC101060796 (1442.426 kb)		Name: ALR/Alpha; Class: Satellite; Family: centr			
Acute UD	PA34	ISLA on p24+ cells	43	7	59841435	F	Upstream: LOC102724116 (1752.85 kb); Downstream: LOC101060796 (1150.467 kb)		Name: ALR/Alpha; Class: Satellite; Family: centr			
Acute UD	PA34	ISLA on p24+ cells	43	7	60749732	F	Upstream: LOC102724116 (2661.147 kb); Downstream: LOC101060796 (242.17 kb)		Name: ALR/Alpha; Class: Satellite; Family: centr			
Acute UD	PA34	ISLA on p24+ cells	44	6	55130485	R	Upstream: FAM83B (185.386 kb); Downstream: HCRTR2 (43.788 kb)		Name: L2; Class: LINE; Family: L2			
Acute UD	PA34	ISLA on p24+ cells	45	3	49729348	F	IP6K1	Opposite	NA			
Acute UD	PA34	ISLA on p24+ cells	46	17	76185105	F	RNF157	Opposite	NA			
Acute UD	PA35	ISLA on p24+ cells	47	4	168496007	F	Upstream: DDX60L (15.492 kb); Downstream: PALLD (1.057 kb)		NA			
Acute UD	PA35	ISLA on p24+ cells	48	16	3803414	R	CREBBP	Same	Name: AluSg4; Class: SINE; Family: Alu			
Acute UD	PA35	ISLA on p24+ cells	49	16	36499419	F	Upstream: LOC101929923 (397.214 kb); Downstream: ANKRD26P1 (9969.918 kb)		Name: ALR/Alpha; Class: Satellite; Family: centr			
Acute UD	PA35	ISLA on p24+ cells	49	16	37359240	F	Upstream: LOC101929923 (1257.035 kb); Downstream: ANKRD26P1 (9110.097 kb)		Name: ALR/Alpha; Class: Satellite; Family: centr			
Acute UD	PA35	ISLA on p24+ cells	50	17	76054811	F	SRP68	Opposite	NA			
Acute UD	PA35	ISLA on p24+ cells	51	22	40457680	R	MKL1	Same	NA			
Acute UD	PA35	ISLA on p24+ cells	52	4	6704414	R	Upstream: S100P (7.244 kb); Downstream: MRFAP1L1 (3.287 kb)		Name: Tigger17a; Class: DNA; Family: TcMar-Tigger			
Acute UD	PA35	ISLA on p24+ cells	53	15	44435400	R	CTDSPL2	Opposite	Name: AluSx; Class: SINE; Family: Alu			
Acute UD	PA35	ISLA on p24+ cells	54	16	37178089	F	Upstream: LOC101929923 (1075.884 kb); Downstream: ANKRD26P1 (9291.248 kb)		Name: ALR/Alpha; Class: Satellite; Family: centr			
Acute UD	PA35	ISLA on p24+ cells	55	2	26307969	R	LOC105374334	Opposite	Name: AluY; Class: SINE; Family: Alu			
Acute UD	PA35	ISLA on p24+ cells	56	17	28942130	R	PHF12	Mixed	NA			
Acute UD	PA35	ISLA on p24+ cells	56	17	28942130	R	LOC101927018		NA			
Acute UD	PA35	ISLA on p24+ cells	57	15	44600210	R	SPG11	Same	Name: L1ME4a; Class: LINE; Family: L1			
Acute UD	PA35	ISLA on p24+ cells	58	20	28904021	R	Upstream: LOC100289097 (301.183 kb); Downstream: LOC102723316 (176.23 kb)		Name: ALR/Alpha; Class: Satellite; Family: centr			
Acute UD	PA35	ISLA on p24+ cells	59	17	76200623	R	RNF157	Same	Name: L1MB2; Class: LINE; Family: L1			
Acute UD	PA35	ISLA on p24+ cells	60	22	49860482	F	ALG12	Mixed	Name: L1MC4; Class: LINE; Family: L1			
Acute UD	PA35	ISLA on p24+ cells	60	22	49860482	F	ZBED4					
Acute UD	PA35	ISLA on p24+ cells	61	19	21404930	R	ZNF493	Opposite	NA		x	
Acute UD	PA35	ISLA on p24+ cells	62	19	49585603	F	PRRG2	Same	Name: AluSx; Class: SINE; Family: Alu			
Acute UD	PA35	ISLA on p24+ cells	63	17	80733686	F	RPTOR	Same	NA			
Acute UD	PA35	ISLA on p24+ cells	64	13	49772990	R	KPNA3	Same	Name: L1ME1; Class: LINE; Family: L1			
Acute UD	PA35	ISLA on p24+ cells	65	6	42778948	F	LOC105375062	Same	Name: AluSx1; Class: SINE; Family: Alu			
Acute UD	PA35	ISLA on p24+ cells	66	19	34416113	F	PDCD2L	Same	Name: AluSx; Class: SINE; Family: Alu			
Acute UD	PA35	ISLA on p24+ cells	67	1	167920124	F	MPC2	Opposite	NA			
Acute UD	PA35	ISLA on p24+ cells	68	12	102045219	F	CCDC53	Opposite	NA			
Acute UD	PA35	ISLA on p24+ cells	69	10	69272218	R	HK1	Opposite	Name: Charlie4a; Class: DNA; Family: hAT-Charlie			
Acute UD	PA35	ISLA on p24+ cells	70	15	60606642	F	RORA;	Mixed	NA			
Acute UD	PA35	ISLA on p24+ cells	70	15	60606642	F	RORA-AS1		NA			
Acute UD	PA35	ISLA on p24+ cells	71	14	91401188	R	CCDC88C	Same	Name: L1ME4a; Class: LINE; Family: L1			
Acute UD	PA35	ISLA on p24+ cells	72	18	49098048	F	DYM	Opposite	NA			
