# Supplementary material for: Evolution of the HIV-1 integration site landscape and inducible reservoir in early-treated people
Source: PLoS Pathog. 2025 Nov 25;21(11):e1013702. doi: 10.1371/journal.ppat.1013702 (PMC12646413; doi:10.1371/journal.ppat.1013702)
Supplement: S3 Table — NA = not available; ISLA = Integration Site Loop Amplification; FLIPS = Full-Length Individual Proviral Sequencing; HXB2 = subtype B HIV-1 reference genome. (PDF) [file ppat.1013702.s010.pdf]

Supplementary Table 3. List of primers used in this manuscript

Assay	Amplicon	HXB2 coordinates	Forward/reverse	Round	Primer	Sequence (5' to 3')
RPP30 qPCR	RPP30	NA	Forward	1	Forward	AGATTTGGACCTGCGAGCG
			Reverse	1	Reverse	GAGCGGCTGTCTCCACAAGT
			Probe	1	Probe	TTCTGACCTGAAGGCTCTGCGCG
ISLA 3'		8948	Forward	1	up3.2	CCAATGCTGATTGTGCCTGGCTAGAAGCA
		NA	Forward	2	deca1.U5	TCAAGTAGTGTGTGCCCGTCTGTNNNNNNNNNN
		9553	Forward	3	RF2	AGACCAGATCTGAGCCTGGGAGCTCTCTG
		9595	Forward	4	RF1	CCCACTGCTTAAGCCTCAATAAAGCTTGCCTTG
		9626	Forward	5	1.U5	TGAGTGCTTCAAGTAGTGTGTGCCCGTCTGT
		9647	Forward	6	2.U5	GCCCGTCTGTTGTGTGACTCTGGTAACTAGAGAT
ISLA 5'		651	Reverse	1	UTR.629.R	CCCTGTTCGGGCGCCACTGCTA
		NA	Reverse	2	decaU3R.3	GTTCTGCCAATCAGGGAAGTAGCCTTGTGTGTNNNNNNNNNN
		160	Reverse	3	U3R.1	GGCTCAACTGGTACTAGCTTGAAGCACCATCCAAAG
		118	Reverse	4	U3R.2	GGATATCTGATCCCTGGCCCTGGTGTGTAGTT
		89	Reverse	5	U3R.3	GTTCTGCCAATCAGGGAAGTAGCCTTGTGTGT
		51	Reverse	6	U3R.4	CCCACAGATCAAGGATATCTTGTCT
Rainbow	RU5	518-647	Forward	1	RU5 Fwd	TTAAGCCTCAATAAAGCTTGCC
			Reverse	1	RU5 Rvd	GTTCGGGCGCCACTGCTAGA
			Probe	1	RU5 probe	/56ROXN/CCAGAGTCACACAACAGACGGGCACA/3IAbRQSp/
	psi	692-797	Forward	1	psi Fwd	CAGGACTCGGCTTGCTGAAG
			Reverse	1	psi Rvd	GCACCCATCTCTCTCCTTCTAGC
			Probe	1	psi probe	/56-FAM/TTTTGGCGT/ZEN/ACTCACCAGT/3IABkFQ/
	env	7736-7851	Forward	1	env Fwd	AGTGGTGCAGAGAGAAAAAAGAGC
			Reverse	1	env Rvd	GTCTGGCCTGTACCGTCAGC
			Probe	1	env dark probe	CC+TTAGGTTCTTAGG+AGC
			Probe	1	env probe	/5HEX/CCTTGGGTT/ZEN/CTTGGGA/3IABkFQ/
	gag	1300-1377	Forward	1	gag Fwd	ATGTTTTCAGCATTATCAGAAGGA
			Reverse	1	gag Rvd	TGCTTGATGTCCCCCCACT
			Probe	1	gag probe	/5Cy5/CCACCCCAC/TAO/AAGATTTAAACACCATGCTAA/3IAbRQSp/
	pol	2536-2662	Forward	1	pol Fwd	GCACTTTAAATTTTCCCATTAGTCCTA
			Reverse	1	pol Rvd	CAAATTTCTACTAATGCTTTTATTTTTTC
			Probe	1	pol probe	/5ATTO550N/AAGCCAGGAATGGATGGCC/3IAbRQSp/
Half genome	Left-half	544-5968	Forward	1	F544	TTAAGCCTCAATAAAGCTTGCCTTGAG
			Reverse	1	R5968	TGTCTYCKCTTCTTCCTGCCATAG
		581-5783	Forward	2	F581	GTGTGCCCGTCTGTTGTGTGACTC
			Reverse	2	R5783	AATGCCTATTCTGCTATGTYGACACC
	Right-half	5066-9665	Forward	1	F5066alt1	TATGGAAAACAGATGGCAGGTGMTGRT
			Reverse	1	R9665	GTCTGAGGGATCTCTAGWTACCAGA
		5088-9602	Forward	2	F5088alt1	GATTGTGTGGCARGTAGACAGRATG
			Reverse	2	R9602	CAAGGCAAGCTTTATTGAGGCTTAAS
2-amplicon for 5' half	Frag1	634-3500	Forward	2	634(+)	AGTGGCGCCCGAACAGGGAC
			Reverse	2	3500(-)	CTATTAAGTATTTTGATGGGTCATAA
	Frag2	1870-5248	Forward	2	1870(+)	GAGTTTTGGCTGAGGCAATGAG
			Reverse	2	5248(-)	TCTCCTGTATGCAGACCCCA
FLIPS	Round 1	623-9662	Forward	1	BLouterF	AAATCTCTAGCAGTGGCGCCCGAACAG
	Round 1		Reverse	1	BLouterR	TGAGGGATCTCTAGTTACCAGAGTC
	Round 2	646-9650	Forward	2	275F	ACAGGGACCTGAAAGCGAAAG
	Round 2		Reverse	2	280R	CTAGTTACCAGAGTCACACAACAGACG
