# Supplementary material for: Evolution of the HIV-1 integration site landscape and inducible reservoir in early-treated people
Source: PLoS Pathog. 2025 Nov 25;21(11):e1013702. doi: 10.1371/journal.ppat.1013702 (PMC12646413; doi:10.1371/journal.ppat.1013702)
Supplement: S4 Table — For 3 individuals (PA35, PA02 and PC10) we made patient-specific primers and/or probe for the indicated amplicons. (PDF) [file ppat.1013702.s011.pdf]

Supplementary Table 4. Participant-specific primer and probes.

Participant	Amplicon	Forward/Reverse	Primer	Sequence (5' to 3')
PA35	*env*	Forward	env Fwd	AGTGGTGCAGAGAGAGAAAAGAGC
		Probe	dark env probe	CC+TTAGGCTCTTAGG+AGC
		Probe	env probe	/5HEX/CCTTGGGCT/ZEN/CTTGGGA/3IABkFQ/
PA02	*psi*	Probe	psi probe	/56-FAM/TTTCAGCGT/ZEN/ACTCACCGTT/3IABkFQ/
	pol	Forward	pol Fwd	GTACTTTAAATTTTCCCATTAGTCCTA
		Reverse	pol Rvd	CAAATTTCTGTTAATGCTTTTATTTTCTC
PC10	*env*	Forward	env Fwd	AGTGGTACAGAGAGAAAAAAGAGC
		Probe	env probe	/5HEX/CATTGGGTT/ZEN/CTTGGGA/3IABkFQ/
		Probe	dark env probe	CA+TTAGGTTCTTAGG+AGC
