# Supplementary material for: Evolution of the HIV-1 integration site landscape and inducible reservoir in early-treated people
Source: PLoS Pathog. 2025 Nov 25;21(11):e1013702. doi: 10.1371/journal.ppat.1013702 (PMC12646413; doi:10.1371/journal.ppat.1013702)
Supplement: S5 Table — Each line shows the acquired information for one sorted p24 + cell. Summary of PCR success for qPCR (RPP30 and HIV), integration site and near full-length proviral sequencing. Green color = positive PCR result, Red color = negative PCR result, Grey color = not attempted. LH = left half; RH = right half; Frag = fragment;/ = not available; N = naïve T cell; CM = central memory T cell; EM = effector memory T cell; IS = integration site. (PDF) [file ppat.1013702.s012.pdf]

Supplementary Table 5: Phenotype, HIV-1 integration site and proviral sequence of p24+ cells.

Participant	Plate	Well #	Phenotype	IS	Defect	RPP30+	LTR+	ISLA 3'	ISLA5'	LH	RH	Frag1	Frag2
PA02	TJL	B3	EM	/									
PA02	TJL	B4	EM	/									
PA02	TJL	B5	EM	Upstream: CD84 (1.02 kb); Downstream: SLAMF1 (27.575 kb)	Incomplete								
PA02	TJL	B6	N	/									
PA02	TJL	B7	CM	RORA	PSI/MSD								
PA02	TJL	B8	CM	/									
PA02	TJL	B9	CM	CD72	PSI/MSD								
PA02	TJL	C2	CM	/	Incomplete								
PA02	TJL	C3	EM	*HIV loop*									
PA02	TJL	C4	EM	ADCY7	PSI/MSD								
PA02	TJL	C5	EM	Chr6: 31553386									
PA02	TJL	C6	EM	Upstream: HEMK1 (8.912 kb); Downstream: LOC105377084 (1.021 kb)	PSI/MSD								
PA02	TJL	C7	EM	SKAP1	PSI/MSD								
PA02	TJL	C8	CM	*HIV loop*	Large deletion								
PA02	TJL	C9	EM	Upstream: LOC102724360 (10.667 kb); Downstream: SUGP2 (33.385 kb)									
PA02	TJL	D2	EM	/									
PA02	TJL	D3	EM	/									
PA02	TJL	D4	CM	PPP1R16B	PSI/MSD								
PA02	TJL	D5	EM	/									
PA02	TJL	D6	CM	Chr19: 3542749	Incomplete								
PA02	TJL	D7	EM	*HIV loop*	PSI/MSD								
PA02	TJL	D8	EM	Chr6: 31553386	PSI/MSD								
PA14	TNE	B3	CM	*HIV loop*									
PA14	TNE	B4	CM	*HIV loop*	Incomplete								
PA14	TNE	B5	EM	*HIV loop*									
PA14	TNE	B6	CM	ZFY	PSI/MSD								
PA14	TNE	B7	EM	RPA1	PSI/MSD								
PA14	TNE	B8	CM	*HIV loop*									
PA14	TNE	B9	CM	Upstream: RPL12P42 (10.562 kb); Downstream: CACNA1A (37.006 kb)	PSI/MSD								
PA14	TNE	B10	CM	UNK	Incomplete								
PA14	TNE	C2	N	SKI	Incomplete								
PA14	TNE	C3	Unknown	/									
PA14	TNE	C4	EM	PELI1	Incomplete								
PA14	TNE	C5	Unknown	*HIV loop*	Incomplete								
PA14	TNE	C6	EM	*HIV loop*									
PA14	TNE	C7	Unknown	/									
PA14	TNE	C8	EM	*HIV loop*	Incomplete								
PA14	TNE	C9	EM	Chr7: 100206687	Incomplete								
PA14	TNE	D2	EM	TGFB1	Incomplete								
PA14	TNE	D3	CM	R3HDM2	Incomplete								
PA14	TNE	D4	N	*HIV loop*	Incomplete								
PA14	TNE	D5	CM	/									
PA14	TNE	D6	Unknown	*HIV loop*	Incomplete								
PA14	TNE	D7	Unknown	USP24	PSI/MSD								
PA14	TNE	D8	EM	RBL1	Incomplete								
PA14	TNE	D9	CM	*HIV loop*									
PA14	TNE	E2	CM	/									
PA14	TNE	E3	EM	/									
PA14	TNE	E4	CM	LOC105378945	PSI/MSD								
PA14	TNE	E5	EM	/									
PA14	TNE	E6	EM	*HIV loop*									
PA14	TNE	E7	N	TGDS	PSI/MSD								
PA14	TNE	E8	EM	*HIV loop*									
PA14	TNE	E9	CM	DENND5A	Incomplete								
PA14	TNE	F2	Unknown	/									
PA14	TNE	F3	EM	*HIV loop*	PSI/MSD								
PA14	TNE	F4	CM	*HIV loop*	PSI/MSD								
PA14	TNE	F5	CM	Upstream: SAMHD1 (36.35 kb); Downstream: RBL1 (8.156 kb)	Incomplete								
PA14	TNE	F6	CM	CDC16	Incomplete								
PA14	TNE	F7	EM	*Chr2: 191197284*	Incomplete								
PA14	TNE	F8	CM	*HIV loop*	Incomplete								
PA14	TNE	F9	CM	SF1									
PA14	TNE	G2	CM	NEK7	Incomplete								
PA14	TNE	G3	CM	VARS	Incomplete								
PA14	TNE	G4	CM	/									
PA14	TNE	G5	CM	*HIV loop*	Incomplete								
PA14	TNE	G6	CM	/									
PA14	TNE	G7	CM	FAM134B	Incomplete								
PA14	TNE	G8	EM	ERN1	Incomplete								
PA14	TNE	G9	CM	ZBTB7B									
PA34	TJM	B3	CM	SEC14L1	PSI/MSD								
PA34	TJM	B4	CM	AKIRIN1	Inversion								
PA34	TJM	B5	CM	Upstream: LOC100421674 (0.291 kb); Downstream: TOP2A (6.392 kb)	Incomplete								
PA34	TJM	B6	CM	/									
PA34	TJM	B7	CM	/									
PA34	TJM	B8	CM	Chr21:8241204, 8424228, 8468788	Intact								
PA34	TJM	B9	CM	*HIV loop*									
PA34	TJM	C2	CM	/									
PA34	TJM	C3	CM	/									
PA34	TJM	C4	CM	Chr22: 22992297	PSI/MSD								
PA34	TJM	C5	CM	Chr21: 8228149, 8411148, 8455295	Intact								
PA34	TJM	C6	EM	PTPRM									
PA34	TJM	C7	CM	ZNF484	Incomplete								
PA34	TJM	C8	EM	*HIV loop*	PSI/MSD								
PA34	TJM	C9	EM	Chr 7: 59549476, 59841435, 60749732	Intact								
PA34	TJM	D2	CM	/									
PA34	TJM	D3	CM	/									
PA34	TJM	D4	EM	/									
PA34	TJM	D5	CM	/									
PA34	TJM	D6	CM	/									
PA34	TJM	D7	CM	/									
PA34	TJM	D8	CM	Upstream: FAM83B (185.386 kb); Downstream: HCRTR2 (43.788 kb)	Incomplete								
PA34	TJM	D9	CM	IP6K1	PSI/MSD								
PA34	TJM	E9	EM	RNF157	PSI/MSD								
PA35	TJK	B10	CM	/									
PA35	TJK	F10	CM	Chr17: 28942130									
PA35	TJK	F2	CM	SPG11									
PA35	TJK	F3	CM	/									
PA35	TJK	F8	CM	*HIV loop*	PSI/MSD								
PA35	TJK	G2	CM	*HIV loop*									
PA35	TJK	G4	CM	PRRG2	PSI/MSD								
PA35	TJK	H2	CM	MPC2	Incomplete								
PA35	TJK	H3	CM	CCDC53									
PA35	TJK	H5	CM	Chr15: 60606642	Incomplete								

PA35	TJK	H8	CM	CCDC88C	PSI/MSD
PA35	TJK	B4	CM	CREBBP	
PA35	TJK	B5	CM	*HIV loop*	PSI/MSD
PA35	TJK	B6	CM	*HIV loop*	Intact
PA35	TJK	B7	CM	Chr16: 36499419, 37359240	Large deletion
PA35	TJK	C2	CM	MKL1	Intact
PA35	TJK	C4	CM	Upstream: S100P (7.244 kb); Downstream: MRFAP1L1 (3.287 kb)	
PA35	TJK	D5	CM	/	Incomplete
PA35	TJK	D8	CM	/	
PA35	TJK	E3	CM	/	Intact
PA35	TJK	E5	CM	pstream: LOC101929923 (1075.884 kb); Downstream: ANKRD26P1 (9291.248 kb)	
PA35	TJK	E8	CM	LOC105374334	Incomplete
PA35	TJK	C9	CM	/	PSI/MSD
PA35	TJK	E10	EM	/	
PA35	TJK	H10	EM	PDCD2L	
PA35	TJK	C10	N	SRP68	PSI/MSD
PA35	TJK	D10	N	/	Incomplete
PA35	TJK	G10	N	/	
PA35	TJK	F5	N	RNF157	
PA35	TJK	F7	N	Chr22: 49860482	Incomplete
PA35	TJK	F9	N	ZNF493	
PA35	TJK	G3	N	/	
PA35	TJK	G6	N	RPTOR	PSI/MSD
PA35	TJK	G7	N	/	
PA35	TJK	G8	N	KPNA3	Large deletion
PA35	TJK	H4	N	HK1	Intact
PA35	TJK	H6	N	/	
PA35	TJK	H7	N	/	
PA35	TJK	H9	N	DYM	PSI/MSD
PA35	TJK	B3	N	Upstream: DDX60L (15.492 kb); Downstream: PALLD (1.057 kb)	PSI/MSD
PA35	TJK	B8	N	/	PSI/MSD
PA35	TJK	B9	N	/	
PA35	TJK	C3	N	*HIV loop*	
PA35	TJK	C5	N	/	PSI/MSD
PA35	TJK	C6	N	/	
PA35	TJK	C7	N	*HIV loop*	
PA35	TJK	C8	N	/	
PA35	TJK	D3	N	/	
PA35	TJK	D4	N	*HIV loop*	Incomplete
PA35	TJK	D6	N	/	
PA35	TJK	D7	N	CTDSPL2	
PA35	TJK	D9	N	/	
PA35	TJK	E2	N	/	
PA35	TJK	E6	N	/	
PA35	TJK	E7	N	*HIV loop*	
PA35	TJK	E9	N	*HIV loop*	
PA35	TJK	E4	Td	/	Incomplete
PA35	TJK	F4	Unknown	pstream: LOC100289097 (301.183 kb); Downstream: LOC102723316 (176.23 k	re stopcodon/frameshift
PA35	TJK	F6	Unknown	/	
PA35	TJK	G5	Unknown	/	
PA35	TJK	G9	Unknown	LOC105375062	PSI/MSD
PA35	TJK	D2	Unknown	/	Intact
