# Supplementary material for: Evolution of the HIV-1 integration site landscape and inducible reservoir in early-treated people
Source: PLoS Pathog. 2025 Nov 25;21(11):e1013702. doi: 10.1371/journal.ppat.1013702 (PMC12646413; doi:10.1371/journal.ppat.1013702)
Supplement: Attachment — Submitted filename: Response to Reviewers.pdf [file ppat.1013702.s015.pdf]

Answers to the reviewers' comments

Part II – Major Issues: Key Experiments Required for Acceptance

Reviewer #1: None.

We thank the reviewer for the careful assessment of our manuscript. We are pleased that only minor revisions were requested and have addressed these on page 4.

Reviewer #2:

1) The acute cohort includes nine individuals, with only five followed longitudinally to UD+5. The chronic cohorts are cross-sectional, and only three chronic participants were included for integration site analysis due to primer bias. These limitations restrict the ability to draw strong conclusions about differences between acute and chronic initiation or about longitudinal trends. The authors should temper generalizations and clearly frame this as a small but valuable exploratory dataset.

As an example:

The results section states that "integration into heterochromatin regions is already detectable early after ART initiation in the acute cohort and significantly increases over time on ART." While centromeric or satellite integrations were seen, the absolute numbers are small and enrichment is driven by single individuals. A more accurate representation would be that "such integrations are detectable in some acute participants and enriched in certain individuals over time, consistent with the possibility of early selection for proviruses in heterochromatin."

We thank the reviewer for this thoughtful comment. We acknowledge this limitation and have noted it in the Discussion: *Future studies with larger cohorts will be necessary to validate and extend our findings, as these are based on a relatively small sample size (lines 415-416 track changes file).*

To provide a more accurate representation, we have clarified in the Results the number of individuals in whom these integrations were detected (lines 165-168 and 171-174 *track changes file*). Furthermore, S2 Fig shows in detail the number of integrations in centromeric and KRAB-ZNF genes on chr19 for each individual. Moreover, we also clarified in the Discussion (lines 339-341 *track changes file*): *Furthermore, in three out of five participants of the Acute UD+5 cohort, a selection for proviruses integrated into centromeric/satellite DNA after five years on ART was observed.*

The sentence referred to by the reviewer was revised (lines 204-207 *track changes file*): *Interestingly, integration into repressive chromatin regions, including centromeric DNA and KRAB-ZNF gene clusters on chr19, is already detectable early after ART initiation in more than half of the participants of the acute cohort (5/8) and significantly increases over time on ART in several individuals (3/5).*

Finally, we tempered the concluding statement in the Discussion (lines 350-353 *track changes file*): *Although based on a small yet valuable exploratory dataset, these results suggest that post-integration selection mechanisms may shape the long-term composition of the reservoir in early-treated individuals and will need to be confirmed in larger cohorts.*

2) The finding that clonal expansion increases over time in early-treated individuals is not unexpected and has been described previously, for example in Coffin et al., JCI Insight 2019. The

novelty here lies in the longitudinal confirmation within an acute cohort, rather than in demonstrating the phenomenon itself. The authors should adjust their framing accordingly.

We agree that clonal expansion in early-treated individuals has been described by Coffin et al. 2019, which we have cited and discussed. We adjusted the framing to emphasize that we build further upon this data, by providing longitudinal evidence of clonal expansion beyond the first year of ART in early-treated individuals (see lines 59, 314-316, 318-319, 323 track changes file).

3) The observation of increasing integration in centromeric or satellite DNA and KRAB-ZNF genes at UD+5 is intriguing, but the absolute numbers are small, enrichment is sometimes driven by single participants. The conclusions about post-integration selection should therefore be presented more cautiously.

We agree that the number of integrations in centromeric/satellite DNA and KRAB-ZNF genes is limited. However, we note that the observed enrichment is not driven by a single participant but is seen in three of the five longitudinally followed individuals. We have revised the text to present these findings more cautiously, now stating (lines 171-177 track changes file): *Interestingly, paired analysis of the five longitudinally followed individuals from the acute cohort revealed an increase in the proportion of integrations in centromeric/satellite DNA with time on ART in three of the five individuals (UD: 1.52%; UD+5: 8.87%; p = 0.001), suggestive of a positive selection of viruses integrated into heterochromatin regions (S2 Fig).*

The use of Tat-LNP with PMA is an important technical strength and clearly improves detection sensitivity, but this is an extreme stimulation condition, and it is unclear whether proviruses detected under these conditions—especially those integrated in heterochromatin—would be inducible in vivo or relevant for rebound. The authors should add stronger caveats re: this point.

We agree with the reviewer that the PMA/Tat-LNP condition represents a strong in vitro stimulation unlikely to reflect reactivation in vivo. We acknowledge this in the Discussion (lines 395-401 track changes file): *Interestingly, we could detect inducible intact proviruses integrated in centromeric regions, suggesting that a block-and-lock profile may be reversible. However, given that in vitro reactivation with maximal stimulation unlikely reflects reactivation in vivo, it is uncertain whether these proviruses would participate in viral rebound. Nevertheless, PMA/Tat-LNP appears to be highly effective in enabling the detection and further characterization of the translation-competent reservoir in individuals with small reservoir sizes, marking an important achievement for in vitro studies.*

The detection of inducible proviruses integrated in centromeric regions aligns with prior observations[1,2]. It has also been shown that replication-competent viruses located in centromeric regions can be recovered by viral outgrowth assays (qVOA)[1], although such assays also use maximal stimulation and unlikely reflects reactivation in vivo.

Reviewer #3: Though not experimental, this reviewer would like to address data analysis questions in key figures.

1. Figure 2, integration site clones: Figure 2 focuses focus on the bulk IS landscape of UD, UD+5 vs control. Limited sampling at some conditions (7, 20, 26) limits the ability to capture a complete picture of clonality; however, remarkably, the detection of clones with intra/inter-sample, does speak to an apparently large clone size such that they may be detected at limited sampling. Rather than or in addition to percent clone/total IS, a calculation of oligoclonality or clonal diversity many allow for a quantitative comparison across acute, acute+5, and control.

We thank the reviewer for the suggestion to calculate the oligoclonality index (OCI) to provide a quantitative comparison across the cohorts. Therefore, we calculated the OCI for the Acute UD, Acute UD+5 and Chronic UD cohorts and added this as Fig 2C. In the Results we included the following (lines 146-155 track changes file): *To provide a quantitative measure of clonal expansion, we calculated the oligoclonality index (OCI)[3] for each individual (Fig 2C). Values range between 0 and 1, where 0 indicates complete heterogeneity (each integration site is detected only once), and 1 indicates complete homogeneity (all integration sites come from a single clone). At UD, the OCI was higher in the chronic cohort compared to the acute cohort (medians = 0.029 and 0.061 in Acute UD and Chronic UD, respectively; p = 0.28). Within the acute cohort, the OCI increased from UD to UD+5 among the five longitudinally followed individuals (paired analysis: medians = 0.028 and 0.082 in Acute UD and Acute UD+5, respectively; p = 0.0625); suggesting an increase in clonal expansion.*

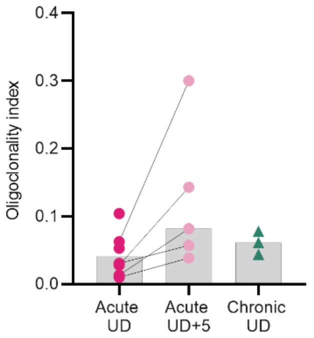

Fig 2(C) Oligoclonality index for each individual of the Acute UD (n = 8), Acute UD+5 (n = 5) and Chronic UD cohort (n = 3). Grey bars depict median values. Lines connect paired values for the longitudinally followed individuals in the acute cohort.

2. Figure 6, phylogenetic diversity: The authors note a lack of phylogenetic diversity in acute (acute+5) proviral sequences relative to chronic. This is consistent with the field and supported for acute in F6. Is there any quantification for diversity from the chronic cohort, and comparing something like average pairwise distance (APD) between the groups? Such analysis quantification would lend support to “Our findings confirm that early initiation of ART limited the total size and phylogenetic diversity 270 of the viral reservoir compared to ART initiation during chronic infection.”

We thank the reviewer for this suggestion. This analysis is included in the manuscript. Specifically, S1F–G Figs show the average genetic distance across cohorts and timepoints. As described in the Results (*Clinical and virological characteristics of the participants*, lines 124-130 track changes file): *In addition, we evaluated the genetic viral diversity among individuals for all cohorts and timepoints (S1F-I Figs). At UD the average intra-individual genetic diversity was lower in the acute cohort compared to the chronic cohort (medians = 1.7 bp and 46.1 bp in Acute UD and Chronic UD, respectively; p = 0.07; S1F Fig). No further sequence diversification was observed during prolonged suppressive ART in the acute (p = 0.13; S1G-H Figs) cohort.*

3. Figure 4: The subset conclusion is supported by Figure 4C; however, 4D at current does not seem to contribute to conclusions, comparing %p24 +/- by Fisher’s exact, which shows more p24+ in EM and less in Naïve. Suggest showing 4D longitudinal, ie %p24+ Acute vs Acute+5, Chronic vs Chronic+x. Can be done non-paired due to missing time points.

We understand the confusion and have clarified this in the Results (lines 234-246 track changes file). Fig 4C and 4D convey two different messages. Fig 4C shows the percentages of each subset in the p24- (~total CD4 T cells) population compared to the p24+ population, revealing that p24+ cells are enriched in TEM compared to the total CD4 T cell population. In Figs 4D and S5B we address longitudinal changes in the phenotype of p24+ cells by solely focusing on p24+ cells.

Part III – Minor Issues: Editorial and Data Presentation Modifications
Please use this section for editorial suggestions as well as relatively minor modifications of existing data that would enhance clarity.

Reviewer #1: The paper is well written, the figures clear and of high quality, and the analysis is insightful yet balanced. My principal yet minor concern is that, in multiple instances, it is unclear whether the authors consider integration into peri-centromeric genic DNA, centromeric satellite DNA, "KRAB-ZNF genes on chr19", and KRAB-ZNF genes elsewhere to be equivalent in terms of heterochromatin environment and/or as regions of generalized transcriptional suppression as a basis for enabling persistence of proximally integrated intact proviruses. Some clarification and/or use of more precise language in these instances would be welcome.

We thank the reviewer for this insightful comment. We agree that it is important to clearly distinguish the different genomic regions and their heterochromatin context. Accordingly, we have revised the manuscript to use more appropriate terminology and to explicitly distinguish between the different repressive chromatin regions analyzed, rather than using a broad heterochromatin label (lines 63, 161, 168-170, 204-207, 343 track changes file). For example, instead of '*Integration in heterochromatin regions is already detectable…*', we now report (lines 204-207 track changes file): *Interestingly, integration into repressive chromatin regions, including centromeric DNA and KRAB-ZNF gene clusters on chr19, is already detectable early after ART initiation in more than half of the participants of the acute cohort (5/8) and significantly increases over time on ART in several individuals (3/5).*

Moreover, we added lines 65-69 (track changes file) to frame the different types of repressive chromatin regions described: *Centromeres are heterochromatin domains composed of repetitive, gene-poor DNA and organized into tightly packed nucleosomes that limit accessibility to the transcriptional machinery[28,29]. Moreover, the human heterochromatin protein CBX1 coats large KRAB-ZNF gene clusters on chromosome 19[30], establishing a repressive chromatin environment.*

Reviewer #2: - Many individuals yielded unreportable results with the Rainbow assay (Supplementary Fig. 1). The limitations of the assay in this context should be discussed.

In our study 19/38 (50%), 5/10 (50%), 13/57 (23%) yielded an unreportable intactness result in the Acute UD, Chronic UD and Chronic UD+x cohort, respectively. Signal failure due to subtype diversity and genetic polymorphism at primer or probe binding sites, is a known limitation of the assay. This has been described in previous studies[7,8] and in the original manuscript describing the Rainbow assay[9]. Personalized primer/probe sets, as designed for some individuals of the acute cohort, lead to a signal recovery and detectable intact HIV-1 DNA.

We addressed this limitation in the Methods of the Rainbow assay in lines 461-463 (track changes file).

- The manuscript consistently refers to the “translation-competent reservoir.” For clarity, the authors should define precisely what Tat-LNP HIV-Flow measures and distinguish it from replication-competent virus.

We thank the reviewer for this suggestion. We have defined the translation-competent reservoir and clarified what the HIV-Flow assay measures in the Results (lines 209–210, 213-214 track changes file), and adjusted the wording to distinguish it from the replication-competent reservoir (lines 383–385, 391-392 track changes file).

Reviewer #3: 1. Figure 3, the text says KRAB-ZNF chr19, but figure text says KRAB-ZNF (total?), please clarify.

We thank the reviewer for noting this inconsistency. We have clarified in the Fig 3B legend and text that the data refer specifically to KRAB-ZNF genes on chromosome 19 (page 37 track changes file).

2. Language Note: Take care to distinguish/acknowledge the impact between ‘preferentially integrated’ (which may have high impact at short timepoint) vs selected/persisted (longer timepoint) (see below line numbers)
a. “Analysis of integration in repetitive elements 164 using RepeatMasker showed that proviruses preferentially integrated into long and short 165 interspersed nuclear elements (LINE, SINE) among all cohorts (Acute UD: 41%; Acute UD+5: 166 31%; Chronic UD: 43% of total integration sites; Figure 3D).”
b. “Furthermore, three out of five participants of the Acute UD+5 cohort showed increased 305 integration”

We agree with the reviewer on the importance of distinguishing the different terms and have adapted the sentences in the Results accordingly (lines 186, 339-341 track changes file).

3. Authors sometimes ‘lump’ KRAB-ZNF and centromere/satellite together in text and thematically. Care should be taken to parse the two groups (genic vs non-genic, chromosomal location, etc) and distinguished as such.
a. Figure 6, grouped together in solid green box

We adapted Fig 6 to highlight the distinction between integrations in (peri-) centromeric DNA and KRAB-ZNF genes on chromosome 19, as indicated by two different color boxes (page 41 track changes file).

b. Figure 2 and text lines 149-153, “Notably, proviral integration in centromeric/satellite DNA (0.88%150 of total IS at UD) and KRAB-ZNF genes on chr19 (0.88% of total IS at UD) was already151 detectable in the acute phase of infection (Figure 3B). Moreover, the acute and chronic cohorts 152 showed similar levels of integration within these regions at UD (Acute UD: 1.77% and Chronic153 UD: 3.55%; p = 0.17).”
i. “These regions” unclear KRAB-ZNF or cent/sat or both?

We clarified this point by rephrasing the sentence (lines 168-171 track changes file) to: *Moreover, the acute and chronic cohorts at UD showed similar levels of integration within centromeric/satellite DNA (Acute UD: 0.88% and Chronic UD: 0.51%; p>0.99) and KRAB-ZNF genes on chr19 (Acute UD: 0.88% and Chronic UD: 3.05%; p = 0.07).*

4. Language Note: Conclusion casts a wide net with catch-all "heterochromatin" rather parameters defined in data.
a. "Interestingly, integration into heterochromatin 183 regions is already detectable early after ART initiation in the acute cohort and significantly 184 increases over time on ART.

We thank the reviewer for this comment. We have addressed this point in response to a similar remark from reviewer 1. Specifically, we revised the text to use more precise terminology and to better distinguish between the different repressive chromatin regions analyzed (centromeric/satellite DNA and KRAB-ZNF gene clusters on chromosome 19). These clarifications are now included in lines 63, 161, 168-170, 204-207, 343 (track changes file).

5. Proviral Sequence/Question: It is of note that STIP-SEQ p24+ proviral/IS sequencing resulting in only 17% intact provirus observed. p24 detection in non-replication competent provirus is of note for 2 points (1) defective provirus protein expression and immune activation and (2) LRA studies assume all p24+ represent reservoir/viremia recrudescent provirus, as such, may overestimate underlying reservoir and/or impact of LRA on true reservoir target. Please make complete/incomplete proviral genomes publicly available.

We agree with the reviewer. The HIV-Flow/STIP-Seq assay measures the translation-competent reservoir but does not directly assess replication-competence. In two previous studies[10,11] from our group, we show that only 10% of proviruses retrieved by STIP-Seq are intact and could potentially be replication-competent, while the majority of the proviruses harbor PSI/MSD defects. Importantly, defective proviruses produce viral RNA and proteins, contributing to chronic immune activation[12,13]. Moreover, PSI/MSD defective proviruses have been identified as a source of non-suppressible viremia in ART-treated individuals[14]. Together, these findings underscore the importance of characterizing reservoir cells harboring defective proviruses. We added this to Discussion (lines 391-392 track changes file): *These defective proviruses can contribute to chronic immune activation[36,38] and non-suppressible viremia, but lack evidence on replication-competence[40].*

We have made the proviral genomes publicly available and added the following statement in the Data availability section: HIV-1 sequence data that supports the findings of this study have been deposited in GenBank with the accession codes: PQ286693 - PQ286864 and PQ286865 - PQ286941.

6. Citation of pediatric cohort/reservoir characterization. May note in introduction previous studies that have characterized integration site landscape in pediatric cohorts (cross-section) as 'early/acute,' though not Fiebig as done here.
a. Bale Kearney ISA Children mBio 2021

b. Garcia-Broncano, Kuritzkes, Lichterfeld STM Neonates STM 2019

We thank the reviewer for the suggestion. We have added a sentence in the Introduction acknowledging the previous studies that characterized the integration site landscape in early-treated pediatric cohorts (Bale et al., mBio 2021; Garcia-Broncano et al., Sci Transl Med 2019). The revised text can be seen on lines 56-60.

References

1. Jiang, C., Lian, X., Gao, C., Sun, X., Einkauf, K.B., Chevalier, J.M., Chen, S.M.Y., Hua, S., Rhee, B., Chang, K., et al. (2020). Distinct viral reservoirs in individuals with spontaneous control of HIV-1. Nature *585*, 261–267. 10.1038/s41586-020-2651-8.

2. Jordan, A., Bisgrove, D., and Verdin, E. (2003). HIV reproducibly establishes a latent infection after acute infection of T cells *in vitro*. EMBO J. *22*, 1868–1877. https://doi.org/10.1093/emboj/cdg188.

3. Gillet, N.A., Malani, N., Melamed, A., Gormley, N., Carter, R., Bentley, D., Berry, C., Bushman, F.D., Taylor, G.P., and Bangham, C.R.M. (2011). The host genomic environment of the provirus determines the abundance of HTLV-1–infected T-cell clones. Blood *117*, 3113–3122. https://doi.org/10.1182/blood-2010-10-312926.

4. Thakur, J., Packiaraj, J., and Henikoff, S. (2021). Sequence, Chromatin and Evolution of Satellite DNA. Int. J. Mol. Sci. *22*. 10.3390/ijms22094309.

5. McNulty, S.M., and Sullivan, B.A. (2018). Alpha satellite DNA biology: finding function in the recesses of the genome. Chromosom. Res. *26*, 115–138. 10.1007/s10577-018-9582-3.

6. Vogel, M.J., Guelen, L., de Wit, E., Peric-Hupkes, D., Lodén, M., Talhout, W., Feenstra, M., Abbas, B., Classen, A.-K., and van Steensel, B. (2006). Human heterochromatin proteins form large domains containing KRAB-ZNF genes. Genome Res. *16*, 1493–1504. 10.1101/gr.5391806.

7. Kinloch, N.N., Ren, Y., Conce Alberto, W.D., Dong, W., Khadka, P., Huang, S.H., Mota, T.M., Wilson, A., Shahid, A., Kirkby, D., et al. (2021). HIV-1 diversity considerations in the application of the Intact Proviral DNA Assay (IPDA). Nat. Commun. *12*, 165. 10.1038/s41467-020-20442-3.

8. Simonetti, F.R., White, J.A., Tumiotto, C., Ritter, K.D., Cai, M., Gandhi, R.T., Deeks, S.G., Howell, B.J., Montaner, L.J., Blankson, J.N., et al. (2020). Intact proviral DNA assay analysis of large cohorts of people with HIV provides a benchmark for the frequency and composition of persistent proviral DNA. Proc. Natl. Acad. Sci. *117*, 18692 LP – 18700. 10.1073/pnas.2006816117.

9. Delporte, M., Lambrechts, L., Blomme, E.E., van Snippenberg, W., Rutsaert, S., Verschoore, M., De Smet, E., Noppe, Y., De Langhe, N., De Scheerder, M.-A., et al. (2025). Integrative Assessment of Total and Intact HIV-1 Reservoir by a 5-Region Multiplexed Rainbow DNA Digital PCR Assay. Clin. Chem. *71*, 203–214. 10.1093/clinchem/hvae192.

10. Cole, B., Lambrechts, L., Gantner, P., Noppe, Y., Bonine, N., Witkowski, W., Chen, L., Palmer, S., Mullins, J.I., Chomont, N., et al. (2021). In-depth single-cell analysis of translation-competent HIV-1 reservoirs identifies cellular sources of plasma viremia. Nat. Commun. 10.1038/s41467-021-24080-1.

11. Pardons, M., Cole, B., Lambrechts, L., van Snippenberg, W., Rutsaert, S., Noppe, Y., De Langhe, N., Dhondt, A., Vega, J., Eyassu, F., et al. (2023). Potent latency reversal by Tat RNA-containing nanoparticle enables multi-omic analysis of the HIV-1 reservoir. Nat. Commun. *14*, 8397. 10.1038/s41467-023-44020-5.

12. Pollack, R.A., Jones, R.B., Pertea, M., Bruner, K.M., Martin, A.R., Thomas, A.S., Capoferri, A.A., Beg, S.A., Huang, S.-H., Karandish, S., et al. (2017). Defective HIV-1 Proviruses Are Expressed and Can Be Recognized by Cytotoxic T Lymphocytes, which Shape the Proviral

Landscape. Cell Host Microbe *21*, 494-506.e4. 10.1016/j.chom.2017.03.008.

13. Imamichi, H., Smith, M., Adelsberger, J.W., Izumi, T., Scrimieri, F., Sherman, B.T., Rehm, C.A., Imamichi, T., Pau, A., Catalfamo, M., et al. (2020). Defective HIV-1 proviruses produce viral proteins. Proc. Natl. Acad. Sci. U. S. A. *117*, 3704–3710. 10.1073/pnas.1917876117.

14. White, J.A., Wu, F., Yasin, S., Moskovljevic, M., Varriale, J., Dragoni, F., Camilo-Contreras, A., Duan, J., Zheng, M.Y., Tadzong, N.F., et al. (2023). Clonally expanded HIV-1 proviruses with 5'-leader defects can give rise to nonsuppressible residual viremia. J. Clin. Invest. *133*. 10.1172/JCI165245.
